# Supplementary material for: Klebsiella pneumoniae type VI secretion system-mediated microbial competition is PhoPQ controlled and reactive oxygen species dependent
Source: PLoS Pathog. 2020 Mar 19;16(3):e1007969. doi: 10.1371/journal.ppat.1007969 (PMC7108748; doi:10.1371/journal.ppat.1007969)
Supplement: S1 Table — (DOCX) [file ppat.1007969.s001.docx]

**Table S1: Primers list**

| **Primer Name** |  | **Sequence (5'-3')** |
| --- | --- | --- |
| **FLAG tag and complement Kp52145-∆*phoQ*** | | |
| Prom_PhoPQ_FLAG_R1 |  | TTA TTA CTT GTC GTC ATC GTC TTT GTA GTC ACT CTG TTT ATC TTC CA |
| Kp52_PhoPQ_compF1 |  | ATC CTG ATG GCT GAC AAG GC |
| **Assessing ROS in *E. coli*** | | |
| furrt1 |  | ATC ACC TGA TCT GCC TCG AC |
| furrt2 |  | CGC AGT GAC CGT AAA GAT AGA G |
| soxSrt1 |  | TAT TCA GGA TCT TAT CGC ATG G |
| soxSrt2 |  | CAA GTA CCA CTT TGA ATA GCC T |
| oxyRrt1 |  | GCT ATC TAT GAA GAT CAC CCG T |
| oxyRrt2 |  | GTG ACC ATC TTC CAG CAT CAG |
| oxySrt1 |  | TTT AAC CCT TGA AGT CAC TGC C |
| oxySrt2 |  | CAC GTT GGC TTT AGT TAT TCG AGT |
| katGrt1 |  | TTT GGT TCT AAC TCC GTC CTG |
| katGrt2 |  | TCT TTA ACA AAC TTC TCG TGG G |
| colirrsArt1(HK) |  | AGT GAT AAA CTG GAG GAA GGT G |
| colirrsArt2(HK) |  | GGA CTA CGA CGC ACT TTA TGA G |
| **Site directed mutagenesis of Kp52145 *phoQ*** | | |
| Kp52_PhoPQ_SDM_F2 |  | GTG TGG AGC TGG TTT GTC TA |
| Kp52_PhoPQ_SDM_R2 |  | TCG AAG CTA ACG CTA TAG CC |
| Kp52_PhoQ_Acidic_F2 |  | ATG ACC CAC TCG GTG GCG ATC AAC C |
| Kp52_PhoQ_Acidic_R2 |  | CTG CGC GTT GTT GTT GTT CTG GCG GAT AGC GTC CAG CTG TTC |
| **Cloning VgrG proteins in bacteria** | | |
| VgrG4_F1 |  | GCG CTC GAG ATG GAT ACC TCT TCA ATA ATT A |
| VgrG4 _R1 |  | CGC CTC GAG TCA CTT ACC CAG GCG GTT CAT TTC GAT ATC AGT GTA TTG ACT TTC GTC CTG TTC AA |
| Kp_PBAD_VgrG2_FWD |  | GCA TGA ATT CAT GGA TGT ACA GAA CTT CGA |
| Kp_PBAD_VgrG2_RVR |  | GCA TAA GCT TTT ATT ATG CTC CCT GTC CAT CCG |
| Kp_PBAD_VgrG1_FWD |  | GCA TAG TAC TAT GAG TAG CGT GAA ATC GTT G |
| Kp_PBAD_VgrG1_RVR |  | GCA TTC TAG ATT ATT ATT TCG CCT TAA CCA AGC |
| **Cloning immunity proteins** | | |
| Kp_SelD_FWD |  | GCA TTC TAG AAT GAA AGG GAT AAC GAC ATG |
| Kp_SelD_RVR |  | GCA TGG TAC CTT ATT ACT GGC CAG CTG ACC TGT |
| Kp_SelE_FWD |  | GCA TTC TAG AAT GGC ATG TCT CCT GCA CAG |
| Kp_SelE_RVR |  | GCA TGG TAC CTT ATT ATG CCT CGC TGA CCC GGC |
| **Cloning clpV protein** |  |  |
| Kp52_clpVcomp_F |  | GTT AAC ACG GAT GAT GCG GTG CGC GC |
| Kp52_clpVcomp_R |  | CTA CTC ATC GGA TAT TGT CCG TTA AC |
| **Generating luciferase promoter fusions** | | |
| Kp52_ProTssK_F |  | GAA TTC ATC GCC CGC GGC GGG AAA GC |
| Kp52_ProTssK_R |  | GCT GAA ACT GCT GCG GGA CC |
| ProT6OmpF |  | ATC GCC CGC GGC GGG AAA GC |
| ProT6OmpR |  | GCA TGG ATC CGC TGA AAC TGC TGC GGG ACC |
| ProtssK1_Check_FWD |  | GCT ATC CAT ACT CCA CTT TC |
| ProtssK1_Check_RVR |  | ACG GAC AGG AAC TAT ATC GC |
| pGPL01luc_check_R1 |  | AAC CGG GAG GTA GAT GAG AT |
| pGPL01luc_check_F1 |  | GAA GCG AAG GTT GTG GAT CT |
| ProtssBcheck_F1 |  | GCC TGG TCG ATA CCT ATA TCC |
| ProtssBcheck_F2 |  | ACC AGG TTA CCG TCC ACA T |
| **Cloning VgrG4 truncations in bacteria** | | |
| VgrG4_DUF_UP |  | GCA TGT TAC CAT GGC GTG AGC AAA TTG ATC AGC |
| VgrG4_DUF_DWN |  | GCA TTC TAG ATT ATT AGC TGG CGG GTC CGC TAT AGG |
| VgrG4_DUF_Rhs_UP |  | GCA TGT TAC CAT GTA CTC CGG CCA ATA ATA AGC |
| VgrG4_DUF_Rhs_DWN |  | GCA TTC TAG ATT ATT AGC TGG CGG GTC CGC TAT AGG |
| VgrG4_Vgr_Rhs_UP |  | GCA TGT TAC CGC ATC GCT GGA TGT GGA AGA |
| VgrG4_Vgr_Rhs_DWN |  | GCA TTC TAG ATT ATT ACT GAT CAA TTT GCT CAC GCA |
| VgrG4_COG_UP |  | GCA TGT TAC CAT GTG CAA TGC AGG ATA CCC TGG |
| VgrG4_COG_DWN |  | GCA TTC TAG ATT ATT AGC CAT TAG CCA TCT CCA GCT |
| Kp52_VgrG1flag_F |  | GAA TTC ATG GAT GTA CAG AAC TTC GA |
| Kp52_VgrG1flag_R |  | CTT GTC GTC ATC GTC TTT TAG TCT TAT GCT CCC TGT CCA TCC GGA ATT C |
| Kp52_VgrG2flag_F |  | GAATTCATGGATGTACAGAACTTCGA |
| Kp52_VgrG2flag_R |  | CTT GTC GTC ATC GTC TTT TAG TCT TAT GCT CCC TGT CCA TCC GGA ATT C |
| VgrG4_DUFflag_UP |  | GCA TGT TAC CAT GGC GTG AGC AAA TTG ATC AGC |
| VgrG4_DUFflag_R |  | GCA TTC TAG ATT ATT AGC TGG CGG GTC CGC TAT AGG GAC TAA AAG ACG ATG ACG ACA AG |
| VgrG1_DUFflag_F |  | CCT GAA GCA GAA AAT TGA AC |
| VgrG1_DUFflag_R |  | CTT GTC GTC ATC GTC TTT TAG TCC TGC CAG CGC CTG CTT TCT T |
| Kp52_VgrG4_VSVG_F1 |  | GCG AGA TCT ATG GAT ACC TCT TCA ATA ATT A |
| Kp52_VgrG4_VSVG_R1 |  | CGC CTC GAG TCA CTT ACC CAG GCG GTT CAT TTC GAT ATC AGT GTA TTG ACT TTC GTC CTG TTC AA |
| Kp52_VgrG4 (1-517vsvg) |  | CGC CTC GAG TCA CTT ACC CAG GCG GTT CAT TTC GAT ATC AGT GTA GCC GTA ATC GGT GCT GAC |
| Kp52_VgrG4 (570-899vsvg) |  | GCG AGA TCT GGC AAG ACG CAG CTG AAC |
| **Construction T6SS mutants in *K. pneumoniae*** | | |
| KP_C2_TssL_UP_FWD1 |  | CAG CGA CAG TGA AGA AAT GG |
| KP_ C2_TssL_UP_RVR1 |  | GGA TCC TGT CAT GAC CGC AGA ACC GC |
| KP_ C2_TssL_DWN_FWD1 |  | GGA TCC ACC AGG CGT CAG CAT TAC CT |
| KP_ C2_TssL_DWN_RVR1 |  | TGT CAT GAC CGC AGA ACC GC |
| KP_C1_TssB_UP_FWD1 |  | GCG AAG TCA GCG TTC AGC GT |
| KP_C1_TssB_UP_RVR1 |  | GGA TCC AGC CAT AGC AGT TCC TTT CC |
| KP_C1_TssB_DWN_FWD1 |  | GGA TCC CTT GCC CCG AAA TAA GTG CG |
| KP_C1_TssB_DWN_RVR1 |  | CGG ATA TAT TCC GCA CGA TC |
| KP_C1_TssE_UP_FWD1 |  | CCT TCC GCG AGA AGT TTA TG |
| KP_C1_TssE_UP_RVR1 |  | GGA TCC GGA GCA AGC CTT TCC GTC AG |
| KP_C1_TssE_DWN_FWD1 |  | GGA TCC CCA CAT CAT CCA CCC GCG AC |
| KP_C1_TssE_DWN_RVR1 |  | CCG GTA AAC ACG TTT GGC AT |
| KP_C2_TssE_UP_FWD1 |  | GGA CTG GCG TGG TTT GAA AT |
| KP_C2_TssE_UP_RVR1 |  | CTC GAG TCC CAT TCC AGT CGT TCT CC |
| KP_C2_TssE_DWN_FWD1 |  | CTC GAG GTT ACG TAT AAG TTT GAC TA |
| KP_C2_TssE_DWN_RVR1 |  | GCT GTT GAG GCT GTA GCC AA |
| KP_VgrG4_UpF |  | AAG CCG CTA TCA ATG ACT GG |
| KP_VgrG4_UpR |  | CTC GAG ATC CAT TAT ATT CCT GTT TT |
| KP_VgrG4_DwnF |  | CTC GAG GAC GAA AGT CAA TGA GTC AC |
| KP_VgrG4_DwnR |  | CCG CGG TTT AAG GCG TTT CG |
| Kp52_clpV_UpF1 |  | TCT AGA GGC GAA AGC CGA CCA ATT GC |
| Kp52_clpV_UpR1 |  | GGA TCC TTC CAT GAG ATC CTC TCT TA |
| Kp52_clpV_DwnF1 |  | GGA TCC ATA TCC GAT GAG TAG CGT GA |
| Kp52_clpV_DwnR1 |  | TCT AGA GAT AAC GCT CAT GCC GAA GC |
| Kp52_vgrG2_upF1 |  | TCTGTCAGCAACCGCAAACC |
| Kp52_vgrG2_upR1 |  | GGATCCATCCATCGTGGGCTTCCTTA |
| Kp52_vgrG2_dwnF1 |  | GGATCCGGACAGGGAGCATAATAATG |
| Kp52_vgrG2_dwnR1 |  | TATCTTCCGACAAAGCAGGC |
| Kp52_vgrG1_upF1 |  | TCTAGAAATGAAAGATGAGCAGACGG |
| Kp52_vgrG1_upR1 |  | CTCGAGCACGCTACTCATCGGATATT |
| Kp52_vgrG1_dwnF1 |  | CTCGAGAAATTGAACAACTGCATGGG |
| Kp52_vgrG1_dwnR1 |  | TCTAGAGCCAGATAACCATTATTCGC |
| **Cloning of VSV-G tagged Hcp1 in Kp52145** | | |
| Hcp2467pBAD30_EcoRI_F1 |  | GCG AAT TCA TGC ACG ACA TCA AA |
| Hcp2468pBAD30_HindIII_R1 |  | GCA AGC TTA CTT TCC AAG TCG GTT CAT CTC TAT GTC TGT ATA CGC GGT GGC GCG TTC G |
| **Cloning of VgrG4 and truncations in yeast** | | |
| VgrG4(1-517)-1 |  | TCAGCACCGATTACGGCTGAAAGACGCAGCTGAACC |
| VgrG4 (1-517)-2 |  | GGTTCAGCTGCGTCTTTCAGCCGTAATCGGTGCTGA |
| VgrG4 (518-899)-1 |  | CGGGATCCGGCAAGACGCAGCTGAAC |
| VgrG4 (518-899)-2 |  | GCTCTAGA TCATTGACTTTCGTCCTGTT |
| VgrG4 (518-837)-1 |  | AGCTGGAGATGGCTAATGGCTAAAGCTATCAGATACCCGTTGC |
| VgrG4 (518-837)-2 |  | GCAACGGGTATCTGATAGCTTTAGCCATTAGCCATCTCCAGCT |
| **Cloning VgrGs n yeast** |  |  |
| VgrG1-1 |  | GCTCTAGACATGGATGTACAGAACTTCGA |
| VgrG1-2 |  | GCTCTAGATTATTTCGCCTTAACCAAGC |
| VgrG2-1 |  | GCTCTAGACATGGATGTACAGAACTTCGA |
| VgrG2-2 |  | GCTCTAGA TTATGCTCCCTGTCCATC |
| VgrG4-1 |  | GCTCTAGACATGGATACCTCTTCAATA |
| VgRG4-2 |  | GCTCTAGATCATTGACTTTCGTCCTG |
| **RT-PCR of T6SS genes** |  |  |
| *hcp1*_f3 |  | TGCACGACATCAAAAACCCC |
| *hcp1*_r2 |  | CGCGAAATATGACGGGCAAT |
| *vgrG1*_f2 |  | CCTGCTTTCGGTGAAGGGAT |
| *vgrG1*_r2 |  | GCCCGTGTTTGTCGATATGC |
| *tssM3*_f3 |  | ATGAGCGTCAGCTGGTTGAA |
| *tssM3*_r2 |  | TCTCGATAAAGCCGGTACGC |
