# Supplementary material for: Klebsiella pneumoniae type VI secretion system-mediated microbial competition is PhoPQ controlled and reactive oxygen species dependent
Source: PLoS Pathog. 2020 Mar 19;16(3):e1007969. doi: 10.1371/journal.ppat.1007969 (PMC7108748; doi:10.1371/journal.ppat.1007969)
Supplement: S1 Fig — Analysis of the loci encoding the T6SS in five K. pneumoniae strains: ATCC43816 (A), NTUH-K2044 (B), SGH1- (C), MGH78578 (D), NJST258_1 (E). Each loci is characterized by a distinct vgrG and a set of putative effector(s) and immunity proteins. For each strain, additional t6ss related genes found elsewhere in the genome are indicated. In all panels, the colour code of the genes indicate putative structural genes (purple), hcp (dark green), vgrG (red), effector (denoted as Kpe for K. pneumoniae effectors) (yellow), immunity protein (denoted as Kpi for K. pneumoniae immunity protein) (orange), regulator (green), and PAAR-domain containing protein (grey). (PDF) [file ppat.1007969.s002.pdf]

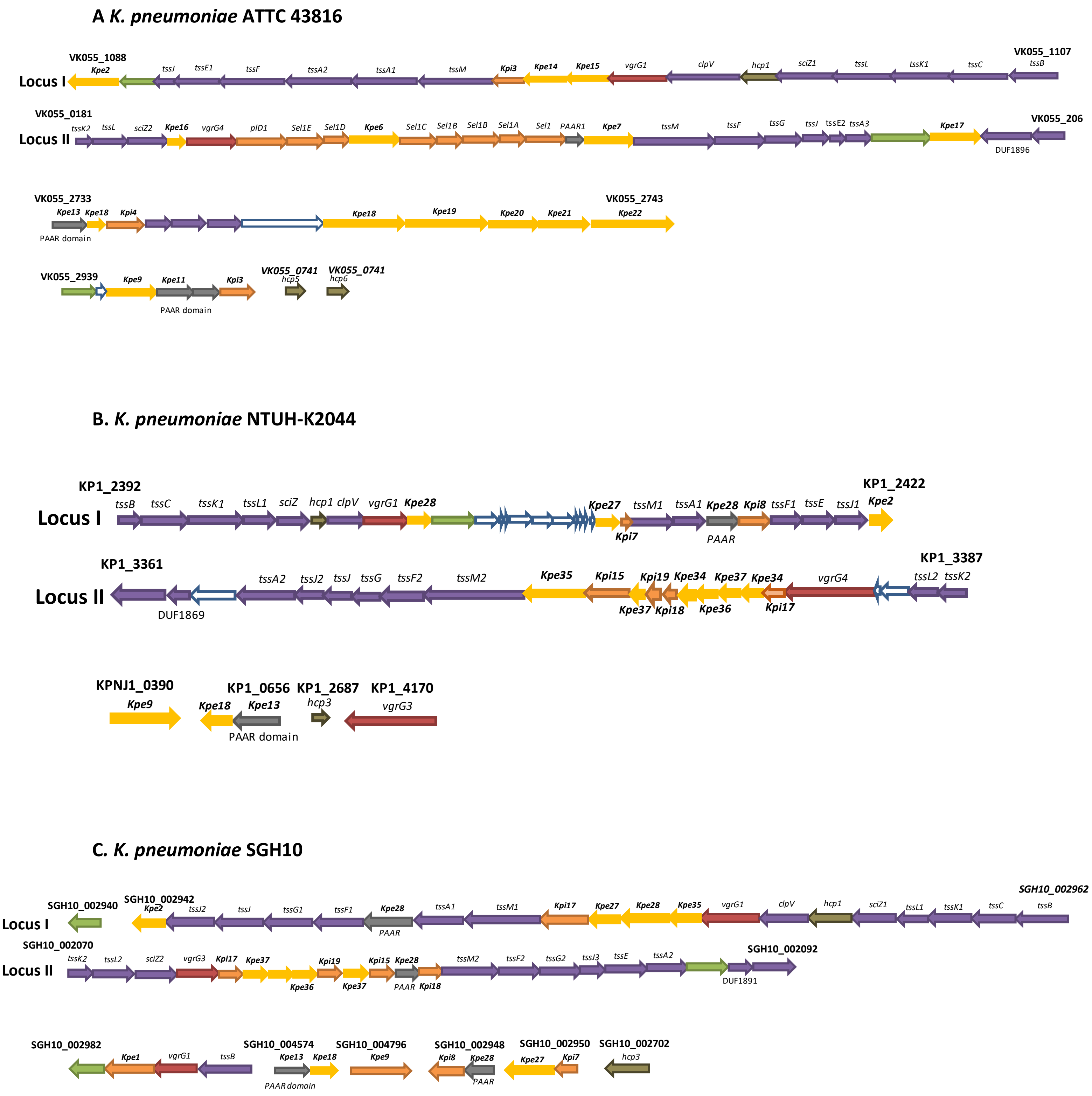

A K. pneumoniae ATTC 43816
Locus I
VK055_1088
Kpe2
tssJ tssE1 tssF tssA2 tssA1 tssM Kpi3 Kpe14 Kpe15 vgrG1 clpV hcp1 sciZ1 tssL tssK1 tssC tssB
VK055_1107
Locus II
VK055_0181
tssK2 tssL sciZ2 Kpe16 vgrG4 plD1 Sel1E Sel1D Kpe6 Sel1C Sel1B Sel1B Sel1A Sel1 PAAR1 Kpe7 tssM tssF tssG tssJ tssE2 tssA3 Kpe17
VK055_206
DUF1896
VK055_2733
Kpe13 Kpe18 Kpi4 Kpe18 Kpe19 Kpe20 Kpe21
VK055_2743
Kpe22
PAAR domain
VK055_2939
Kpe9 Kpe11 Kpi3
PAAR domain
VK055_0741
hcp5
VK055_0741
hcp6
B. K. pneumoniae NTUH-K2044
KP1_2392
Locus I
tssB tssC tssK1 tssL1 sciZ hcp1 clpV vgrG1 Kpe28 Kpe27 tssM1 tssA1 Kpe28 Kpi8 tssF1 tssE tssJ1 Kpe2
KP1_2422
Kpi7
PAAR
KP1_3361
Locus II
DUF1869
tssA2 tssJ2 tssJ tssG tssF2 tssM2 Kpe35 Kpi15 Kpi19 Kpe34 Kpe37 Kpe34 vgrG4 tssL2 tssK2
KP1_3387
Kpe37 Kpi18 Kpe36 Kpi17
KPNJ1_0390
Kpe9
KP1_0656
Kpe18 Kpe13
PAAR domain
KP1_2687
hcp3
KP1_4170
vgrG3
C. K. pneumoniae SGH10
Locus I
SGH10_002940
SGH10_002942
Kpe2 tssJ2 tssJ tssG1 tssF1 Kpe28 tssA1 tssM1 Kpi17 Kpe27 Kpe28 Kpe35 vgrG1 clpV hcp1 sciZ1 tssL1 tssK1 tssC tssB
PAAR
SGH10_002962
SGH10_002070
Locus II
tssK2 tssL2 sciZ2 vgrG3 Kpi17 Kpe37 Kpi19 Kpi15 Kpe28 tssM2 tssF2 tssG2 tssJ3 tssE tssA2
Kpe36 Kpe37 PAAR Kpi18
SGH10_002092
DUF1891
SGH10_002982
Kpe1 vgrG1 tssB
SGH10_004574
Kpe13 Kpe18
PAAR domain
SGH10_004796
Kpe9
SGH10_002948
Kpi8 Kpe28
PAAR
SGH10_002950
Kpe27 Kpi7
SGH10_002702
hcp3

D. *K. pneumoniae* MGH78578

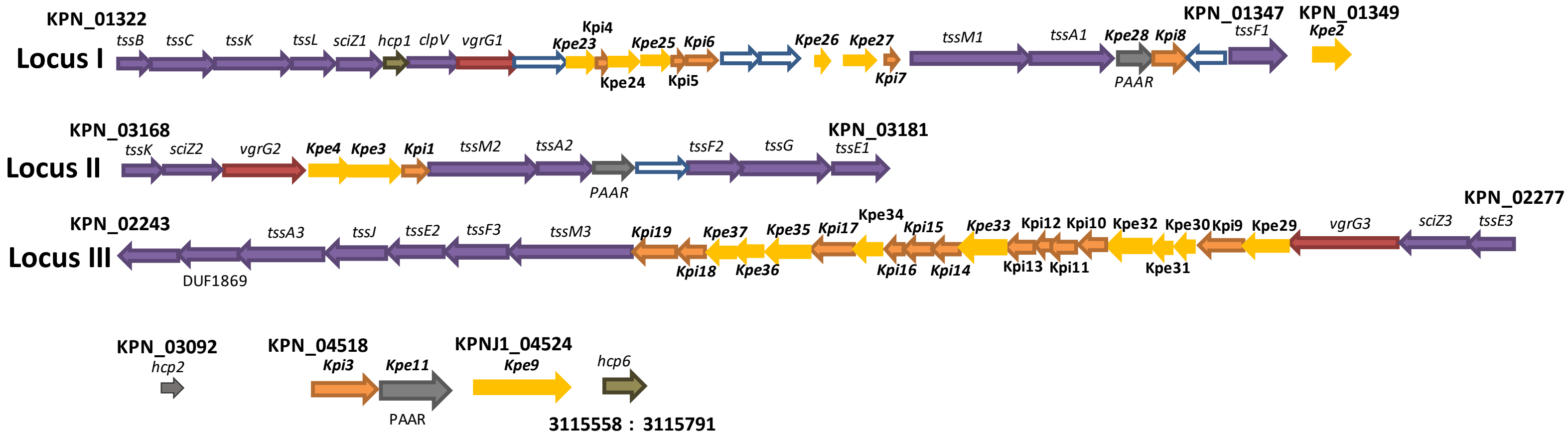

E. *K. pneumoniae* NJST258_1

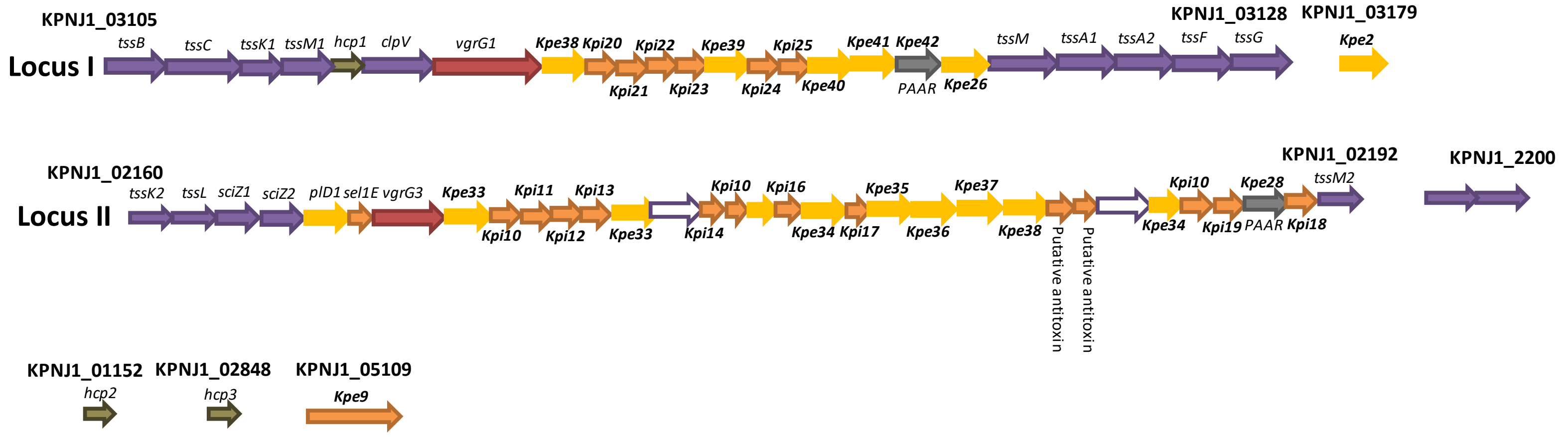

S1 Figure. Predicted T6SS loci in *K. pneumoniae* strains.

Analysis of the gene loci encoding the T6SS in five *K. pneumoniae* strains. Each loci is characterized by a distinct *vgrG* and a set of putative effector(s) and immunity proteins. For each strain, additional *t6ss* related genes found elsewhere in the genome are indicated.

In all panels, the colour code of the genes indicate putative structural genes (purple), *hcp* (dark green), *vgrG* (red), effector (denoted as Kpe for K. pneumoniae effectors) (yellow), immunity protein (denoted as Kpi for K. pneumoniae immunity protein) (orange), regulator (green), and PAAR-domain containing protein (grey).
