# Supplementary material for: Klebsiella pneumoniae type VI secretion system-mediated microbial competition is PhoPQ controlled and reactive oxygen species dependent
Source: PLoS Pathog. 2020 Mar 19;16(3):e1007969. doi: 10.1371/journal.ppat.1007969 (PMC7108748; doi:10.1371/journal.ppat.1007969)
Supplement: S2 Fig — Recovery of E. coli target cell following 6 h incubation with Kp52145, 52145-ΔmanC (ΔmanC), and S. marcescens Db10 (Serratia). The data are presented as means ± the standard deviations (n = 3).****, results are significantly different (P < 0.0001 [two-tailed t test]) from the results for PBS-treated (mock) target cell. (PDF) [file ppat.1007969.s003.pdf]

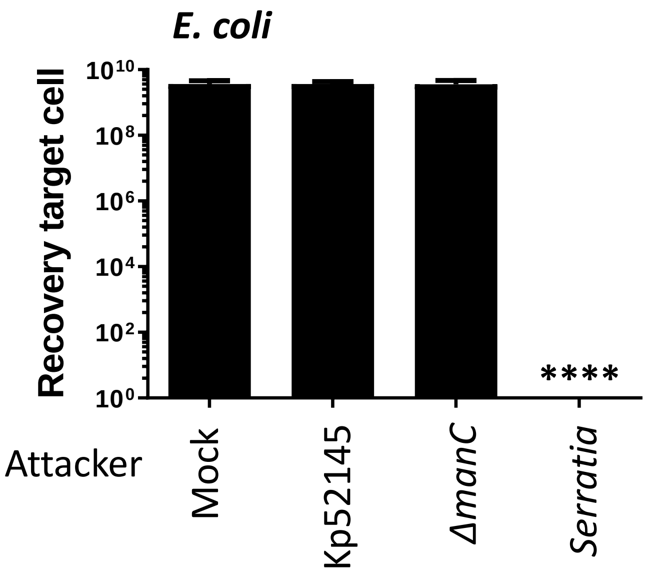

S2 Figure. Lack of bacterial killing when *K. pneumonaie* 52.145 grows in LB.

Recovery of *E. coli* target cell following 6 h incubation with Kp52145, 52145-Δ*manC* (Δ*manC*), and *S. marcescens* Db10 (*Serratia*). The data are presented as means ± the standard deviations (n = 3).****, results are significantly different (P < 0.0001 [two-tailed t test]) from the results for PBS-treated (mock) target cell.
