# Supplementary material for: Klebsiella pneumoniae type VI secretion system-mediated microbial competition is PhoPQ controlled and reactive oxygen species dependent
Source: PLoS Pathog. 2020 Mar 19;16(3):e1007969. doi: 10.1371/journal.ppat.1007969 (PMC7108748; doi:10.1371/journal.ppat.1007969)
Supplement: S3 Fig — (A, B, C) Analysis of the T6SS expression by Kp52145 carrying the transcriptional fusions tssB::lucFF to monitor locus I, tssK2::lucFF to monitor locus II, and sciZ3::lucFF to monitor locus III. At the indicated time points, an aliquot of the culture was taken to measure luciferase levels, and OD600. Luminescence is expressed as relative light units (RLU). The data are presented as means ± the standard deviations (n = 3). (PDF) [file ppat.1007969.s004.pdf]

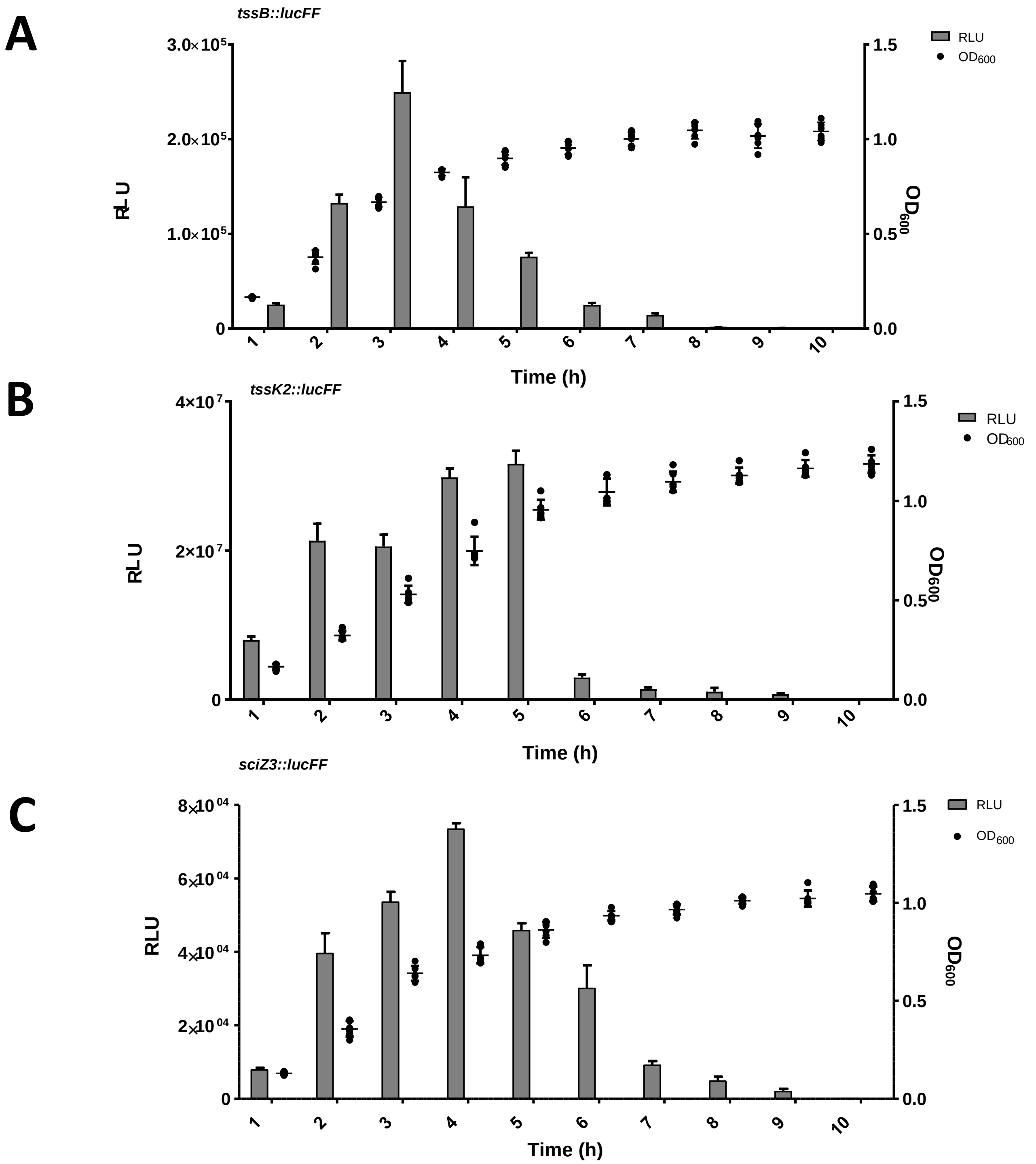

S3 Figure. Effect of growth phase on *K. pneumoniae* 52.145 T6SS expression.

(A, B, C) Analysis of the T6SS expression by Kp52145 carrying the transcriptional fusions *tssB::lucFF* to monitor locus I, *tssK2::lucFF* to monitor locus II, and *sciZ3::lucFF* to monitor locus III. At the indicated time points, an aliquot of the culture was taken to measure luciferase levels, and OD_{600}. Luminescence is expressed as relative light units (RLU). The data are presented as means ± the standard deviations (n = 3)
