# Supplementary material for: Klebsiella pneumoniae type VI secretion system-mediated microbial competition is PhoPQ controlled and reactive oxygen species dependent
Source: PLoS Pathog. 2020 Mar 19;16(3):e1007969. doi: 10.1371/journal.ppat.1007969 (PMC7108748; doi:10.1371/journal.ppat.1007969)
Supplement: S4 Fig — Heat-map analysis of the luciferase levels of the transcriptional fusion tssK2::lucFF (A) and sciZ3::lucFF (B) in the background of the wild-type strain grown until exponential phase at different temperatures (Temp); pH, oxygen tension (1, 15-ml tube incubated vertically shaking at 180 rpm; 2, 15-ml slanted tube (45o) shaking at 180 rpm; 3, 25-ml tube incubated vertically shaking at 180 rpm; 4, 30-ml tube incubated vertically shaking at 180 rpm), and different concentrations of KCl, rhamnose, sucrose, NaCl and the iron chelator 2–2’ bipyridyl. (PDF) [file ppat.1007969.s005.pdf]

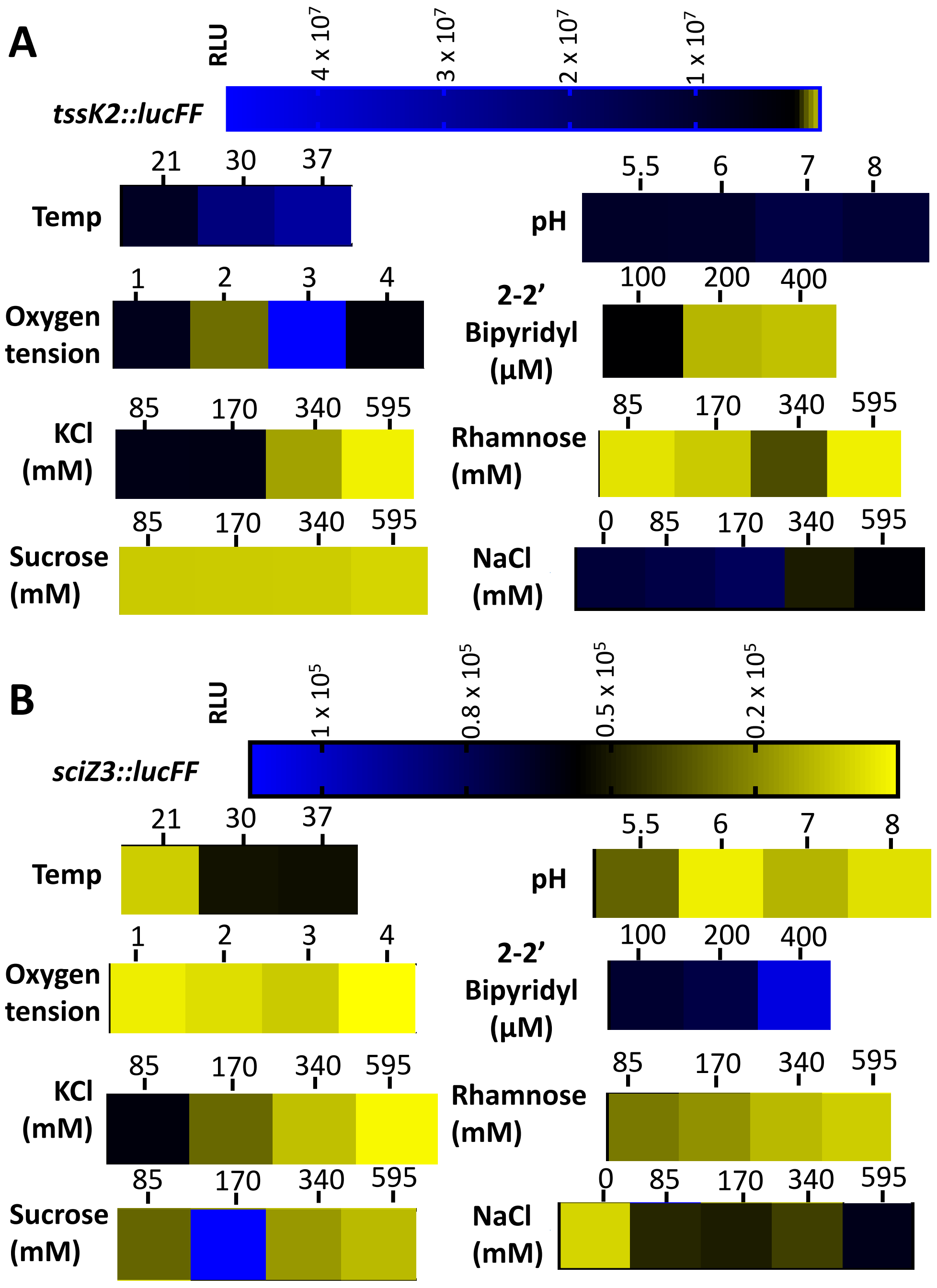

S4 Figure. Regulation of *K. pneumoniae* 52.145 T6SS loci II and III.

Heat-map analysis of the luciferase levels of the transcriptional fusion *tssK2::lucFF* (A) and *sciZ3::lucFF* (B) in the background of the wild-type strain grown until exponential phase at different temperatures (Temp); pH, oxygen tension (1, 15-ml tube incubated vertically shaking at 180 rpm; 2, 15-ml slanted tube (45º) shaking at 180 rpm; 3, 25-ml tube incubated vertically shaking at 180 rpm; 4, 30-ml tube incubated vertically shaking at 180 rpm), and different concentrations of KCl, rhamnose, sucrose, NaCl and the iron chelator 2-2' bipyridyl.
