# Supplementary material for: Klebsiella pneumoniae type VI secretion system-mediated microbial competition is PhoPQ controlled and reactive oxygen species dependent
Source: PLoS Pathog. 2020 Mar 19;16(3):e1007969. doi: 10.1371/journal.ppat.1007969 (PMC7108748; doi:10.1371/journal.ppat.1007969)
Supplement: S5 Fig — T6SS-dependent anti-bacterial activity as determined by recovery of target organism E. coli MG1655 following 6 h co-incubation in LBpH6 (pH6), LBNaCl (NaCl (595 mM) with Kp52145, 52145-ΔclpV (ΔclpV), 52145-ΔmanC (ΔmanC). The data are presented as means ± the standard deviations (n = 3). #, P < 0.0001; n.s. (P > 0.05) not significant differences from the results for mock-treated E. coli; one-way ANOVA Bonferroni correction for multiple comparisons. (PDF) [file ppat.1007969.s006.pdf]

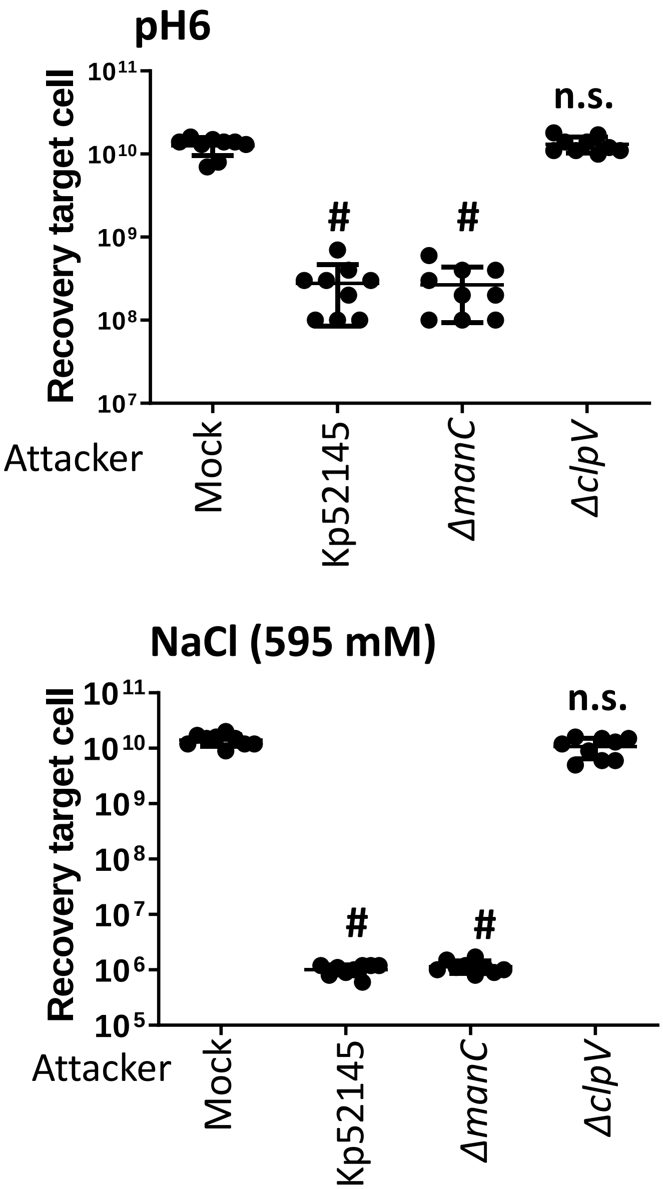

S5 Figure. CPS does not affect *K. pneumoniae* T6SS activity.

T6SS-dependent anti-bacterial activity as determined by recovery of target organism *E. coli* MG1655 following 6 h co-incubation in LB_{pH6} (pH6), LB_{NaCl} (NaCl (595 mM) with Kp52145, 52145-Δ*clpV* (Δ*clpV*), 52145-Δ*manC* (Δ*manC*). The data are presented as means ± the standard deviations (n = 3). #, P < 0.0001; n.s. (P > 0.05) not significant differences from the results for mock-treated *E. coli;* one-way ANOVA Bonferroni correction for multiple comparisons.
