# Supplementary material for: Klebsiella pneumoniae type VI secretion system-mediated microbial competition is PhoPQ controlled and reactive oxygen species dependent
Source: PLoS Pathog. 2020 Mar 19;16(3):e1007969. doi: 10.1371/journal.ppat.1007969 (PMC7108748; doi:10.1371/journal.ppat.1007969)
Supplement: S6 Fig — (A, B) Analysis of the T6SS expression by Kp52145, 52145-Δhfq (hfq), 52145-ΔphoPQGB (phoPQ), 52145-ΔpmrAB (pmrAB), 52145-Δfur (fur), 52145-Δhns (h-ns), 52145-ΔrpoS (rpoS), 52145-ΔrpoN (rpoN), 52145-ΔmgrB (mgrB), 52145-ΔmgrB-ΔphoPQGB (mgrB-phoPQ), 52145-ΔrcsB (rcsB), and 52145-ΔrcsB-ΔphoPQGB (rcsB-phoPQ) carrying the transcriptional fusion tssB::lucFF and grown in LBpH6 (pH6) and LBNaCl (NaCl 595 mM). The data are presented as means ± the standard deviations (n = 3). #, results are significantly different (P < 0.0001) from the results for Kp52145 and for the indicated comparison; ***, results are significantly different (P < 0.001) from the results for Kp52145; n.s. results are not significantly different (P>0.05 001) from the results for Kp52145; one-way ANOVA Bonferroni correction for multiple comparisons. (C, D) Bacterial killing activity mediated by Kp52145, 52145-Δhfq (hfq), 52145-ΔphoPQGB (phoPQ), 52145-ΔpmrAB (pmrAB), 52145-Δfur (fur), 52145-Δhns (h-ns), 52145-ΔrpoS (rpoS), 52145-ΔrpoN (rpoN), 52145-ΔmgrB (mgrB), 52145-ΔmgrB-ΔphoPQGB (mgrB-phoPQ), 52145-ΔrcsB (rcsB), and 52145-ΔrcsB-ΔphoPQGB (rcsB-phoPQ) against E. coli MG1655. Number of recovered target cells following 6 h incubation in LBpH6 (pH6) and LBNaCl (NaCl 595 mM) is indicated. The data are presented as means ± the standard deviations (n = 3). #, results are significantly different (P < 0.0001) from the results for PBS-treated (mock) target cells; one-way ANOVA Bonferroni correction for multiple comparisons. (PDF) [file ppat.1007969.s007.pdf]

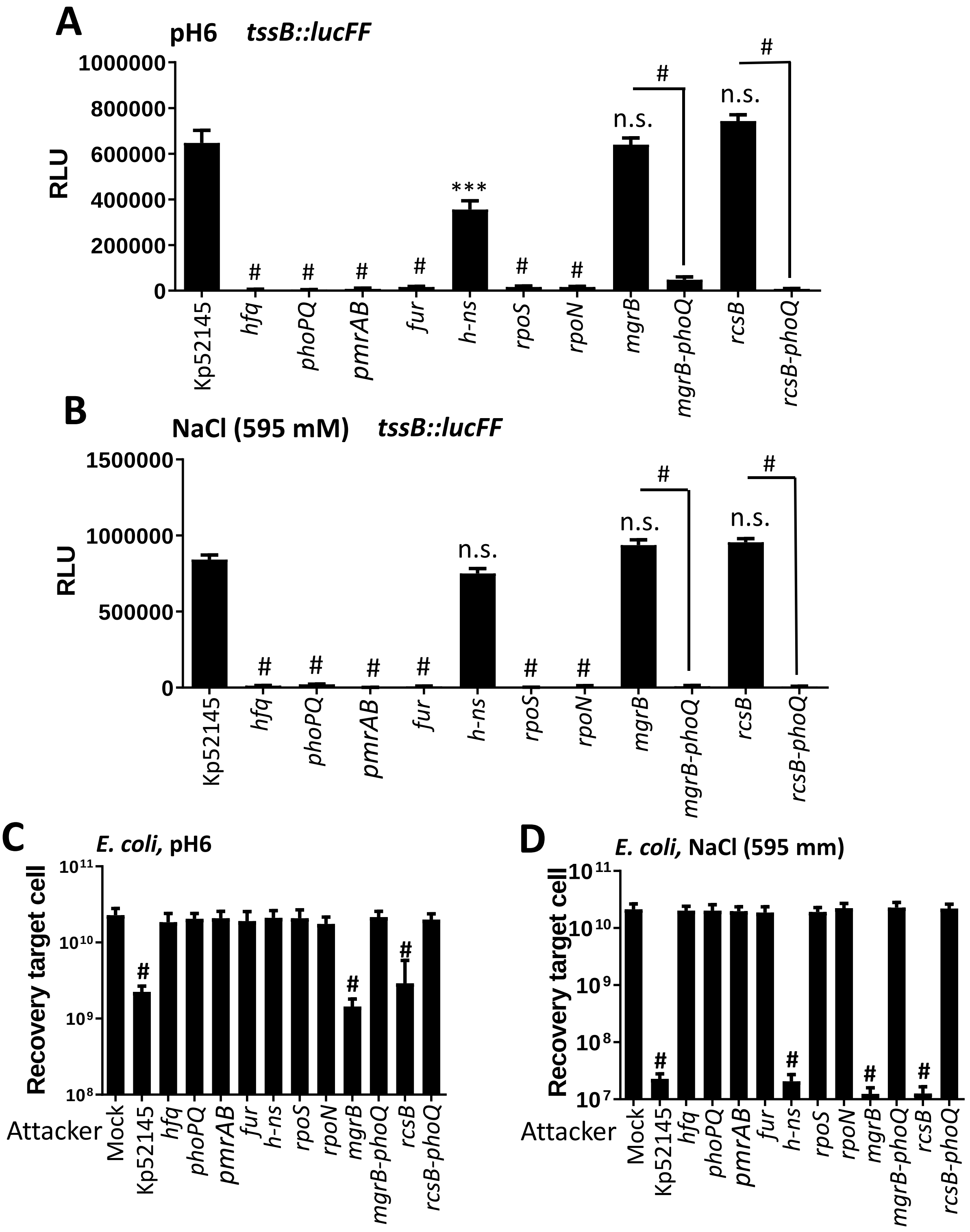

S6 Figure. Identification of transcriptional factors controlling *K. pneumoniae* T6SS expression.

(A, B) Analysis of the T6SS expression by *K. pneumoniae* CIP52.145 (Kp52145), 52145-Δ*hfq* (*hfq*), 52145-Δ*phoPQ*GB (*phoPQ*), 52145-Δ*pmrAB* (*pmrAB*), 52145-Δ*fur* (*fur*), 52145-Δ*hns* (*h-ns*), 52145-Δ*rpoS* (*rpoS*), 52145-Δ*rpoN* (*rpoN*), 52145-Δ*mgrB* (*mgrB*), 52145-Δ*mgrB*-Δ*phoPQ*GB (*mgrB-phoPQ*), 52145-Δ*rcsB* (*rcsB*), and 52145-Δ*rcsB*-Δ*phoPQ*GB (*rcsB-phoPQ*) carrying the

transcriptional fusion *tssB::lucFF* and grown in LB_{pH6} (pH6) and LB_{NaCl} (NaCl 595 mM). The data are presented as means ± the standard deviations (n = 3). #, results are significantly different (P < 0.0001) from the results for Kp52145 and for the indicated comparison; ***, results are significantly different (P < 0.001) from the results for Kp52145; n.s. results are not significantly different (P>0.05 001) from the results for Kp52145; one-way ANOVA Bonferroni correction for multiple comparisons.

(C, D) Bacterial killing activity mediated by *K. pneumoniae* CIP52.145 (Kp52145), 52145-Δ*hfq* (*hfq*), 52145-Δ*phoPQGB* (*phoPQ*), 52145-Δ*pmrAB* (*pmrAB*), 52145-Δ*fur* (*fur*), 52145-Δ*hns* (*h-ns*), 52145-Δ*rpoS* (*rpoS*), 52145-Δ*rpoN* (*rpoN*), 52145-Δ*mgrB* (*mgrB*), 52145-Δ*mgrB*-Δ*phoPQGB* (*mgrB-phoPQ*), 52145-Δ*rcsB (rcsB),* and 52145-Δ*rcsB*-Δ*phoPQGB* (*rcsB-phoPQ*) against *E. coli* MG1655. Number of recovered target cells following 6 h incubation in LB_{pH6} (pH6) and LB_{NaCl} (NaCl 595 mM) is indicated. The data are presented as means ± the standard deviations (n = 3). #, results are significantly different (P < 0.0001) from the results for PBS-treated (mock) target cells; one-way ANOVA Bonferroni correction for multiple comparisons.
