# Supplementary material for: Klebsiella pneumoniae type VI secretion system-mediated microbial competition is PhoPQ controlled and reactive oxygen species dependent
Source: PLoS Pathog. 2020 Mar 19;16(3):e1007969. doi: 10.1371/journal.ppat.1007969 (PMC7108748; doi:10.1371/journal.ppat.1007969)
Supplement: S7 Fig — (A, B, C) T6SS-dependent anti-bacterial activity as determined by recovery of target organisms K. pneumoniae NTUH-K2044 (NTUH-K2044), A. baumannii DSM30011 (DSM30011) following incubation with Kp52145, 52145-ΔclpV (ΔclpV), 52145-ΔphoPQGB (ΔphoPQ), and 52145-ΔphoPQGBCom (ΔphoPQ::phoPQ). #, P < 0.0001; **, P < 0.01; n.s (P > 0.05) not significant differences from the results for PBS-treated (mock) target cell; one-way ANOVA Bonferroni correction for multiple comparisons. (C, D) T6SS-dependent anti-bacterial activity as determined by recovery of target t6ss mutants NTUH-ΔtssB, DSM-ΔtssM following incubation with Kp52145. n.s., (P > 0.05) not significant differences from the results for PBS-treated (mock) target cell. In all panels, the data are presented as means ± the standard deviations (n = 3). (PDF) [file ppat.1007969.s008.pdf]

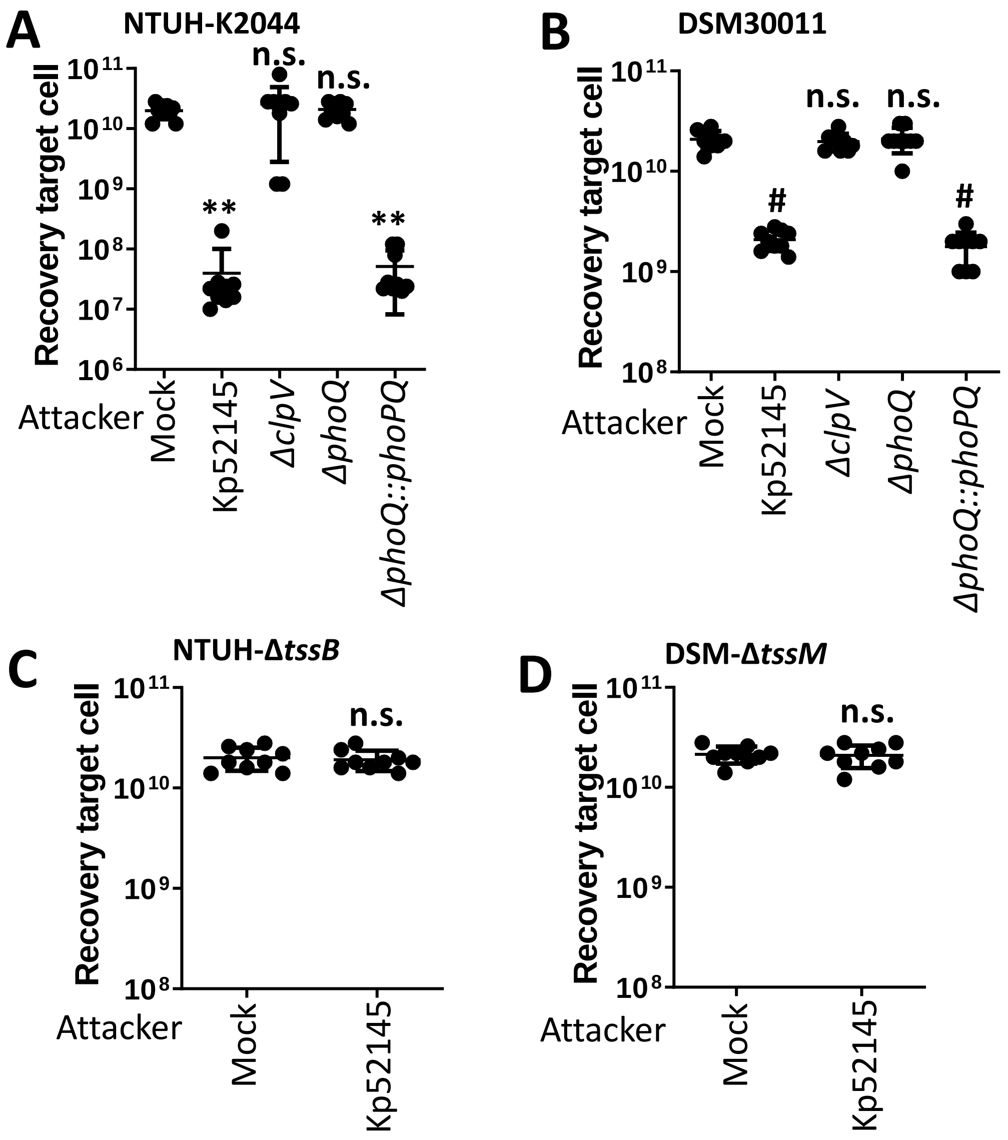

S7 Figure. T6SS-mediated antibacterial competition is fuelled by T6SS active preys.

(A, B, C) T6SS-dependent anti-bacterial activity as determined by recovery of target organisms *K. pneumoniae* NTUH-K2044 (NTUH-K2044), *A. baumannii* DSM30011 (DSM30011) following incubation with Kp52145, 52145-Δ*clpV* (Δ*clpV*), 52145-Δ*phoPQGB* (Δ*phoPQ*), and 52145-Δ*phoPQGB*Com (Δ*phoPQ::phoPQ*). #, $P < 0.0001$; **, $P < 0.01$; n.s ($P > 0.05$) not significant differences from the results for PBS-treated (mock) target cell; one-way ANOVA Bonferroni correction for multiple comparisons.

(C, D) T6SS-dependent anti-bacterial activity as determined by recovery of target *t6ss* mutants NTUH-Δ*tssB*, DSM- Δ*tssM* following incubation with Kp52145. n.s., ($P > 0.05$) not significant differences from the results for PBS-treated (mock) target cell.

In all panels, the data are presented as means ± the standard deviations (n = 3).
