# Supplementary material for: Klebsiella pneumoniae type VI secretion system-mediated microbial competition is PhoPQ controlled and reactive oxygen species dependent
Source: PLoS Pathog. 2020 Mar 19;16(3):e1007969. doi: 10.1371/journal.ppat.1007969 (PMC7108748; doi:10.1371/journal.ppat.1007969)
Supplement: S8 Fig — (A, B, C) T6SS-dependent anti-bacterial activity as determined by recovery of target organisms E. coli MG1655 following incubation with K. pneumoniae ATCC43816 (Kp43816), 43816-ΔtssB (43816-ΔtssB), K. pneumoniae NTUH-K2044 (NTUH), NTUH-ΔtssB (NTUH-ΔtssB), A. baumannii ATCC17978 (17978), 17978-ΔtssM (17978-ΔtssM), A. baumannii DSM30011 (DSM), A. baumannii DSM30011-ΔtssM, (DSM30011-ΔtssM), B. cenocepacia K56-2 (K56-2), and K56-2-ΔatsRΔT6SS (ΔT6SS) in LB. #, results are significantly different (P < 0.0001 [one-way ANOVA Bonferroni for multiple comparisons]) for the indicated comparisons. Mock, PBS-treated target cell. The data are presented as means ± the standard deviations (n = 3). (PDF) [file ppat.1007969.s009.pdf]

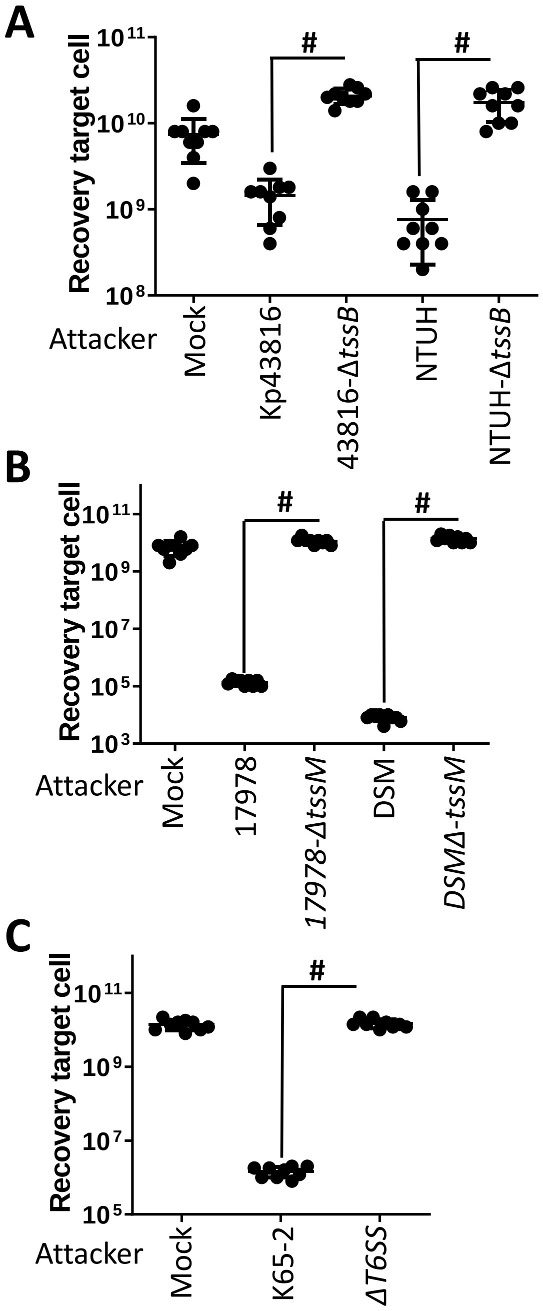

S8 Figure. T6SS-dependent antagonism against *E. coli* prey.

(A, B, C) T6SS-dependent anti-bacterial activity as determined by recovery of target organisms *E. coli* MG1655 following incubation with *K. pneumoniae* ATCC43816 (Kp43816), 43816-Δ*tssB* (43816-Δ*tssB*), *K. pneumoniae* NTUH-K2044 (NTUH), NTUH-Δ*tssB* (NTUH-Δ*tssB*), *A. baumannii* ATCC17978 (17978), 17978-Δ*tssM (*17978-Δ*tssM), A. baumannii* DSM30011 (DSM), *A. baumannii* DSM30011- Δ*tssM*, (DSM30011- Δ*tssM*), *B. cenocepacia* K56-2 (K56-2), and K56-2-Δ*atsR*Δ*T6SS* (Δ*T6SS*) in LB. #, results are significantly different (P < 0.0001 [one-way ANOVA Bonferroni for

multiple comparisons]) for the indicated comparisons. Mock, PBS-treated target cell. The data are presented as means ± the standard deviations (n = 3).
