# Supplementary material for: Klebsiella pneumoniae type VI secretion system-mediated microbial competition is PhoPQ controlled and reactive oxygen species dependent
Source: PLoS Pathog. 2020 Mar 19;16(3):e1007969. doi: 10.1371/journal.ppat.1007969 (PMC7108748; doi:10.1371/journal.ppat.1007969)
Supplement: S9 Fig — (A, B, C) Expression of T6SS by Kp52145, 52145-ΔphoPQGB (ΔphoPQ), and 52145-ΔphoPQGBCom (ΔphoPQ::phoPQ) carrying the transcriptional fusion tssB::lucFF after co-incubation with K. pneumoniae ATCC43816 (Kp43186), K. pneumoniae NTUH-K2044 (NTUH-K2044), A. baumannii ATCC17978 (17978), and B. cenocepacia K56-2 (K56-2). #, results are significantly different (P < 0.0001 [one-way ANOVA Bonferroni for multiple comparisons]) from the results for PBS-treated (mock) Kp52145. In all panels, luminescence is expressed relative light units (RLU) per CFU (x109). The data are presented as means ± the standard deviations (n = 3). (PDF) [file ppat.1007969.s010.pdf]

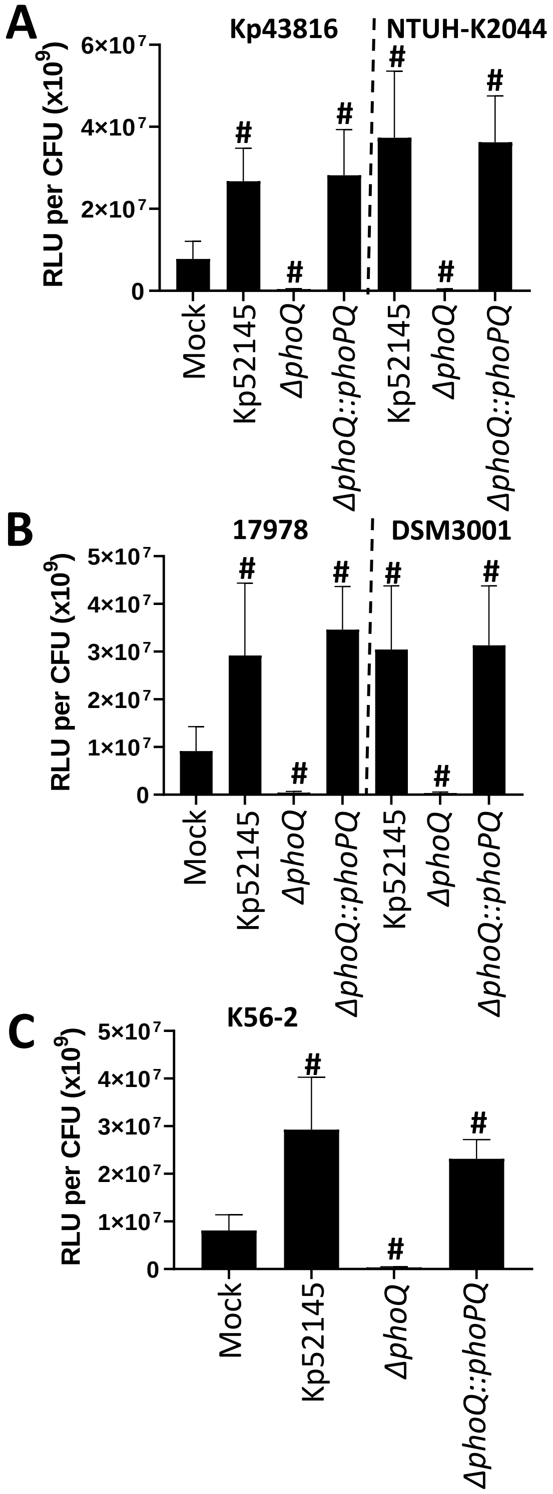

S9 Figure. Co-incubation with bacteria with an active T6SS upregulates *K. pneumoniae* T6SS in a PhoPQ-dependent manner.

(A, B, C) Expression of T6SS by Kp52145, 52145-Δ*phoPQGB* (Δ*phoPQ*), and 52145-Δ*phoPQGB*Com (Δ*phoPQ::phoPQ*) carrying the transcriptional fusion *tssB::lucFF* after co-incubation with *K. pneumoniae* ATCC43816 (Kp43186), *K. pneumoniae* NTUH-K2044 (NTUH-K2044), *A. baumannii* ATCC17978 (17978), and *B. cenocepacia* K56-2 (K56-2). #, results are

significantly different ($P < 0.0001$ [one-way ANOVA Bonferroni for multiple comparisons]) from the results for PBS-treated (mock) Kp52145. In all panels, luminescence is expressed relative light units (RLU) per CFU (x10^9). The data are presented as means ± the standard deviations ($n = 3$).
