# Supplementary material for: Klebsiella pneumoniae type VI secretion system-mediated microbial competition is PhoPQ controlled and reactive oxygen species dependent
Source: PLoS Pathog. 2020 Mar 19;16(3):e1007969. doi: 10.1371/journal.ppat.1007969 (PMC7108748; doi:10.1371/journal.ppat.1007969)
Supplement: S10 Fig — (A, B, C) Expression of T6SS by Kp52145, 52145-ΔphoPQGB (ΔphoPQ), 52145-ΔphoQGBComFLAG (ΔphoPQ::phoPQFLAG), 52145-ΔphoQGBComPhoQ45-190 (ΔphoPQ::phoPQ45-190), and 52145-ΔphoQGBComPhoQGNNNNAQ (ΔphoPQ::phoPQGNNNNAQ) carrying the transcriptional fusion tssB::lucFF after incubation in LB, LBpH6 (pH6), and LBNaCl (NaCl 595 mm). #, results are significantly different (P < 0.0001 [one-way ANOVA Bonferroni for multiple comparisons]) for the indicated comparisons. (D) Western blot analysis using an anti-FLAG antibody demonstrating the presence of PhoQ in cell pellets of 52145-ΔphoQGBComFLAG (phoQ), 52145-ΔphoQGBComPhoQ45-190 (phoQ45-190), and 52145-ΔphoQGBComPhoQGNNNNAQ (phoQGNNNNAQ). Membranes were reprobed with antibody anti RNA Polymerase α. Images are representative of three independent experiments. (PDF) [file ppat.1007969.s011.pdf]

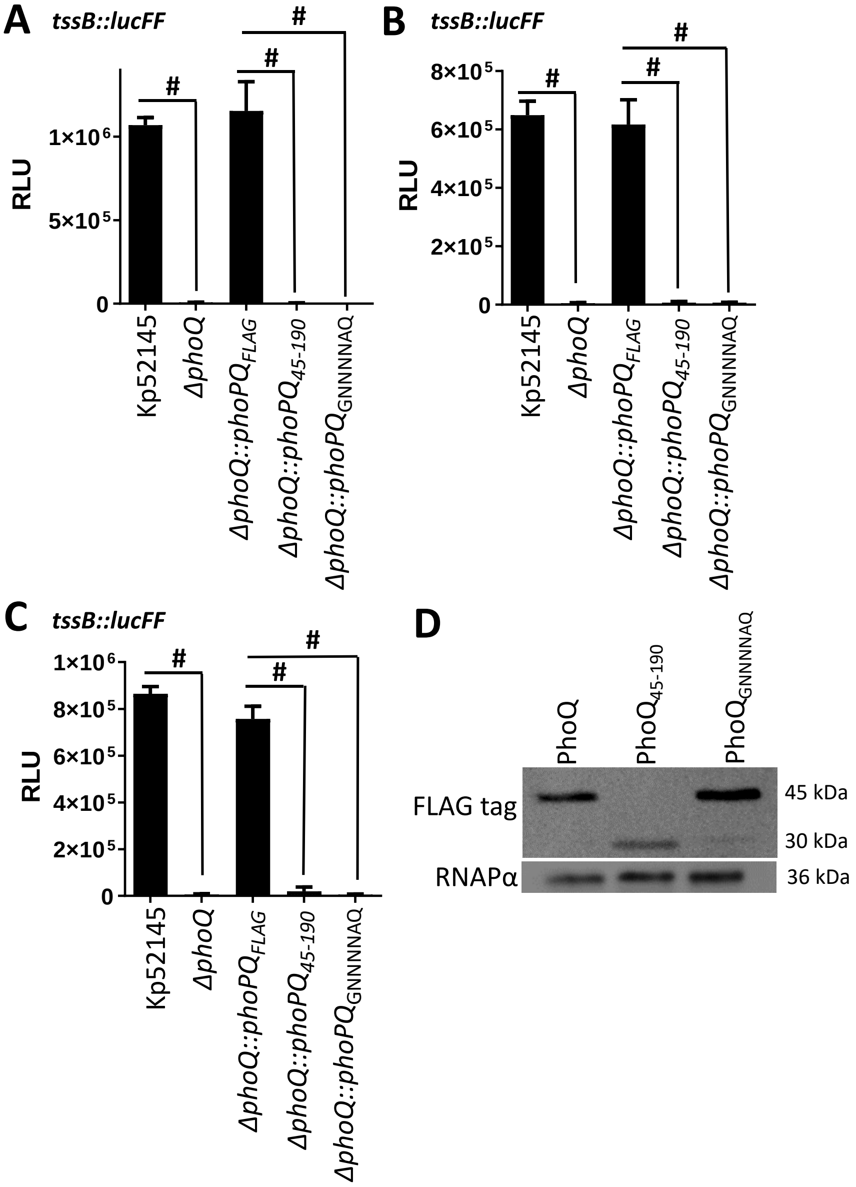

S10 Figure. The periplasmic domain of PhoQ is needed for the regulation of the T6SS.

(A, B, C) Expression of T6SS by Kp52145, 52145-Δ*phoPQGB* (Δ*phoPQ*), 52145-ΔphoQGBComFLAG (Δ*phoPQ::phoPQ*$_{FLAG}$), 52145-ΔphoQGBComPhoQ$_{45\text{-}190}$ (Δ*phoPQ::phoPQ*$_{45\text{-}190}$), and 52145-ΔphoQGBComPhoQ$_{GNNNNAQ}$ (Δ*phoPQ::phoPQ*$_{GNNNNAQ}$) carrying the transcriptional fusion *tssB::lucFF* after incubation in LB, LB$_{pH6}$ (pH6), and LB$_{NaCl}$ (NaCl 595 mm). #, results are significantly different ($P < 0.0001$ [one-way ANOVA Bonferroni for multiple comparisons]) for the indicated comparisons.

(D) Western blot analysis using an anti-FLAG antibody demonstrating the presence of PhoQ in cell pellets of 52145-ΔphoQGBComFLAG (*phoQ*), 52145-ΔphoQGBComPhoQ$_{45\text{-}190}$ (*phoQ*$_{45\text{-}190}$), and

52145-ΔphoQGBComPhoQ$_{GNNNNAQ}$ (*phoQ*$_{GNNNNAQ}$). Membranes were reprobed with antibody anti RNA Polymerase α. Images are representative of three independent experiments.
