# Supplementary material for: Klebsiella pneumoniae type VI secretion system-mediated microbial competition is PhoPQ controlled and reactive oxygen species dependent
Source: PLoS Pathog. 2020 Mar 19;16(3):e1007969. doi: 10.1371/journal.ppat.1007969 (PMC7108748; doi:10.1371/journal.ppat.1007969)
Supplement: S11 Fig — (A, B, C) T6SS-dependent anti-bacterial activity as determined by recovery of target organism E. coli MG1655 following incubation with Kp52145, 52145-ΔclpV (ΔclpV), 52145-ΔvgrG1 (ΔvgrG1), 52145-ΔvgrG1 harbouring pBADVgrG1 (ΔvgrG1/ pBADVgrG1), 52145-ΔvgrG2 (ΔvgrG2), 52145-ΔvgrG4 (ΔvgrG4), 52145-ΔvgrG4 harbouring pBADVgrG4 (ΔvgrG4/pBADVgrG4), 52145-ΔvgrG2-ΔvgrG1 (ΔvgrG2-ΔvgrG1), 52145-ΔvgrG1-ΔvgrG4 (ΔvgrG1-ΔvgrG4), 52145-ΔvgrG2-ΔvgrG4 (ΔvgrG2-ΔvgrG4), 52145-ΔvgrG2- ΔvgrG1-ΔvgrG4 (ΔvgrG2- ΔvgrG1-ΔvgrG4) in LBpH6 (pH6), LBNaCl (NaCl 595 mM), and with sublethal concentration of colistin (0.1 μg/ml) in LB. In all panels, #, P < 0.0001; n.s. (P > 0.05), not significant differences from the results for PBS-treated (mock) target cell; one-way ANOVA Bonferroni for multiple comparisons. The data are presented as means ± the standard deviations (n = 3). (PDF) [file ppat.1007969.s012.pdf]

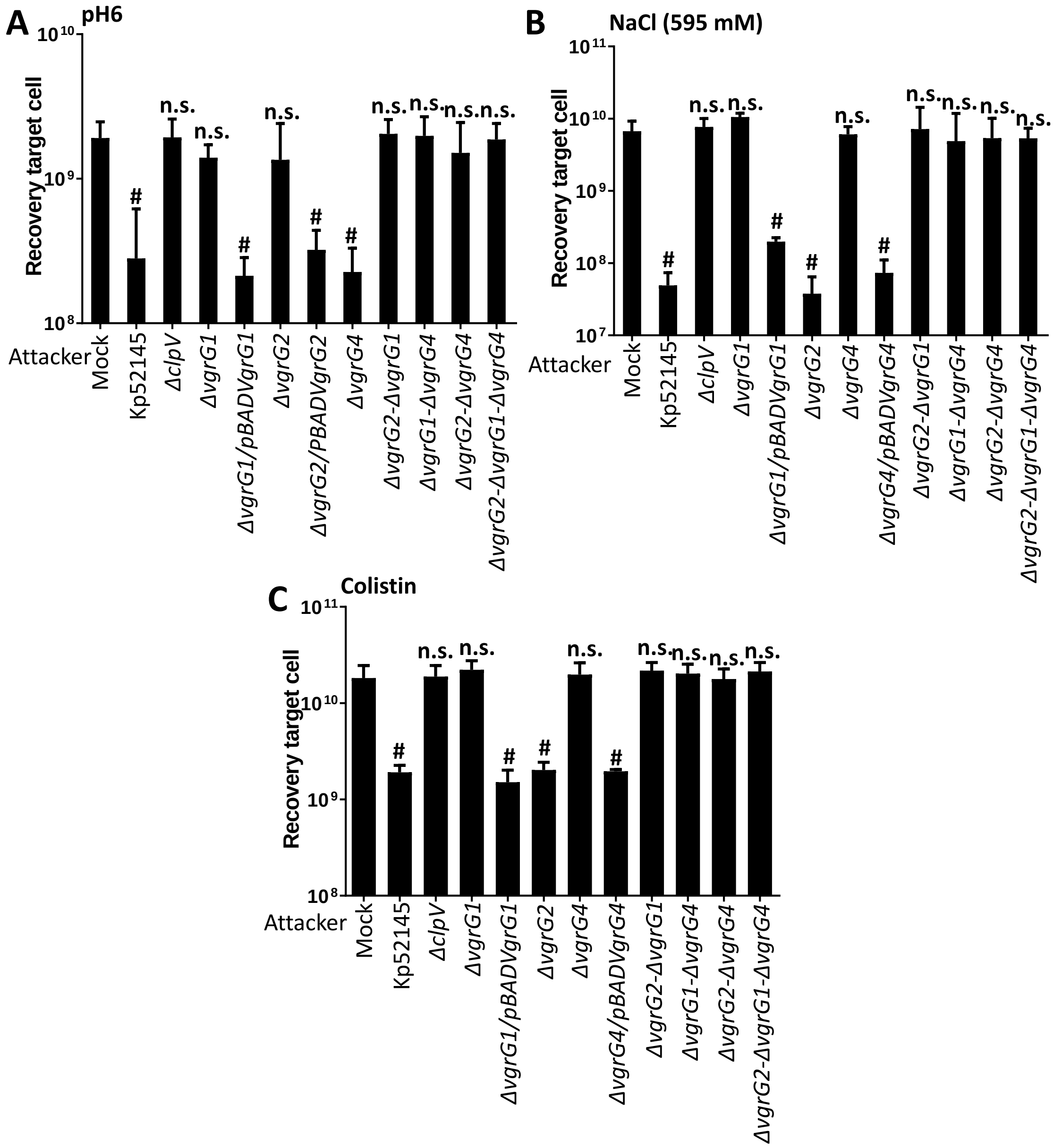

S11 Figure. Role of *K. pneumoniae* T6SS VgrGs in bacterial competition.

(A, B, C) T6SS-dependent anti-bacterial activity as determined by recovery of target organism *E. coli* MG1655 following incubation with Kp52145, 52145-Δ*clpV* (Δ*clpV*), 52145-Δ*vgrG1* (Δ*vgrG1*), 52145-Δ*vgrG1* harbouring pBADVgrG1 (Δ*vgrG1*/ pBADVgrG1), 52145-Δ*vgrG2* (Δ*vgrG2*), 52145-Δ*vgrG4* (Δ*vgrG4*), 52145-Δ*vgrG4* harbouring pBADVgrG4 (Δ*vgrG4*/pBADVgrG4), 52145-Δ*vgrG2*-Δ*vgrG1* (Δ*vgrG2*-Δ*vgrG1*), 52145-Δ*vgrG1*-Δ*vgrG4* (Δ*vgrG1*-Δ*vgrG4*), 52145-Δ*vgrG2*-Δ*vgrG4* (Δ*vgrG2*-Δ*vgrG4*), 52145-Δ*vgrG2*- Δ*vgrG1*-Δ*vgrG4* (Δ*vgrG2*- Δ*vgrG1*-Δ*vgrG4*) in LB_{pH6} (pH6), LBN_{aCl} (NaCl 595 mM), and with sublethal concentration of colistin (0.1 µg/ml) in LB. In all panels, #, $P < 0.0001$; n.s. ($P > 0.05$), not significant differences from the results for PBS-treated (mock) target cell; one-way ANOVA Bonferroni for multiple comparisons. The data are presented as means ± the standard deviations (n = 3).
