# Supplementary material for: Klebsiella pneumoniae type VI secretion system-mediated microbial competition is PhoPQ controlled and reactive oxygen species dependent
Source: PLoS Pathog. 2020 Mar 19;16(3):e1007969. doi: 10.1371/journal.ppat.1007969 (PMC7108748; doi:10.1371/journal.ppat.1007969)
Supplement: S12 Fig — Kaplan–Meier plots showing the per cent survival of G. mellonella over 120 h post-infection with 105 organisms of the following strains: K. pneumoniae NTUH-K2044 (NTHU-K2044), NTHU-ΔtssB, K. pneumoniae ATCC43816 (Kp43816), 43816-(ΔtssB), or PBS. Thirty larvae were infected in each group. Level of significance was determined using the log-rank (Mantel–Cox) test with Bonferroni correction for multiple comparisons where applicable [a = (A) 0.0008; (B) 0.05]. ****, P < 0.0001, ***, P < 0.001 for the indicated comparisons. (PDF) [file ppat.1007969.s013.pdf]

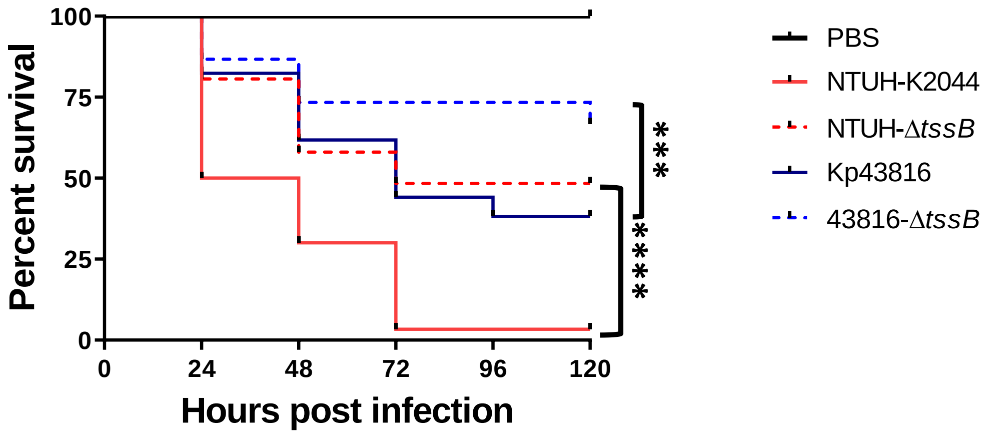

S12 Figure. ***K. pneumoniae*** **T6SS mutants displayed reduced virulence in the** ***G. mellonella*** **infection model.**

Kaplan–Meier plots showing the per cent survival of *G. mellonella* over 120 h post-infection with 10^5 organisms of the following strains: *K. pneumoniae* NTUH-K2044 (NTHU-K2044), NTHU-Δ*tssB*, *K. pneumoniae* ATCC43816 (Kp43816), 43816-(Δ*tssB*), or PBS. Thirty larvae were infected in each group. Level of significance was determined using the log-rank (Mantel–Cox) test with Bonferroni correction for multiple comparisons where applicable [a = (A) 0.0008; (B) 0.05]. ****, P < 0.0001, ***, P < 0.001 for the indicated comparisons.
