# Supplementary material for: Klebsiella pneumoniae type VI secretion system-mediated microbial competition is PhoPQ controlled and reactive oxygen species dependent
Source: PLoS Pathog. 2020 Mar 19;16(3):e1007969. doi: 10.1371/journal.ppat.1007969 (PMC7108748; doi:10.1371/journal.ppat.1007969)
Supplement: S13 Fig — Growth kinetics of K. pneumoniae 51245 (Kp52145), 52145-ΔclpV (ΔclpV), 52145-ΔvgrG1 (ΔvgrG1), 52145-ΔvgrG2 (ΔvgrG2), 52145-ΔvgrG4 (ΔvgrG4), 52145-ΔvgrG2-ΔvgrG1 (ΔvgrG2-ΔvgrG1), 52145-ΔvgrG1-ΔvgrG4 (ΔvgrG1-ΔvgrG4), 52145-ΔvgrG2-ΔvgrG4 (ΔvgrG2-ΔvgrG4), 52145-ΔvgrG2- ΔvgrG1-ΔvgrG4 (ΔvgrG2- ΔvgrG1-ΔvgrG4) in LB broth (LB) (A) and 2% glucose M9 minimal media supplemented with thiamine and MgSO4 (M9-0.2% glucose) (B) over 24 h at 37°C. Values are presented as the mean ± SD of three independent experiments measured in triplicate. (PDF) [file ppat.1007969.s014.pdf]

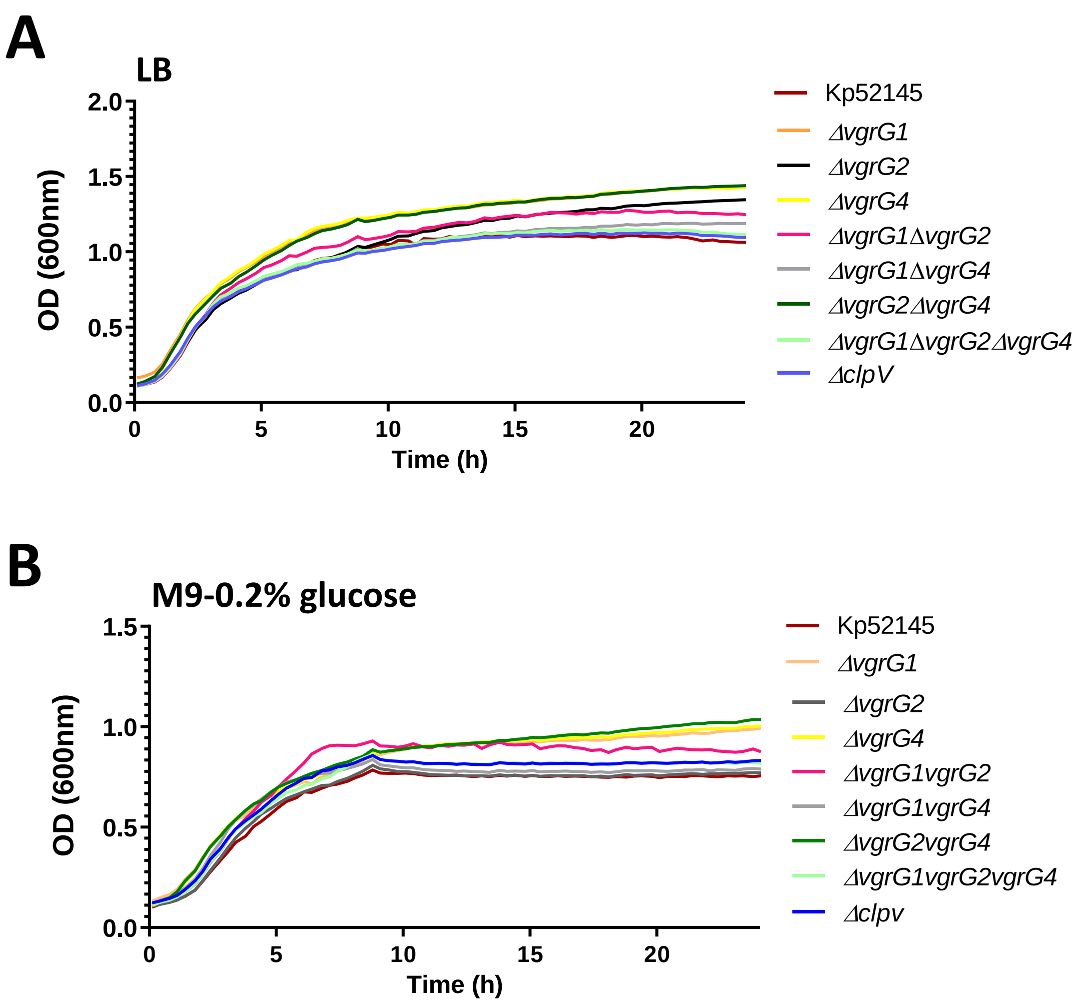

S13 Figure. Growth kinetics of *K. pneumoniae* T6SS mutants.

Growth kinetics of *K. pneumoniae* 51245 (Kp52145), 52145-Δ*clpV* (Δ*clpV*), 52145-Δ*vgrG1* (Δ*vgrG1*), 52145-Δ*vgrG2* (Δ*vgrG2*), 52145-Δ*vgrG4* (Δ*vgrG4*), 52145-Δ*vgrG2*-Δ*vgrG1* (Δ*vgrG2*-Δ*vgrG1*), 52145-Δ*vgrG1*-Δ*vgrG4* (Δ*vgrG1*-Δ*vgrG4*), 52145-Δ*vgrG2*-Δ*vgrG4* (Δ*vgrG2*-Δ*vgrG4*), 52145-Δ*vgrG2*- Δ*vgrG1*-Δ*vgrG4* (Δ*vgrG2*- Δ*vgrG1*-Δ*vgrG4*) in LB broth (LB) (A) and 2% glucose M9 minimal media supplemented with thiamine and MgSO4 (M9-0.2% glucose) (B) over 24 h at 37°C. Values are presented as the mean ± SD of three independent experiments measured in triplicate.
