# Supplementary material for: Klebsiella pneumoniae type VI secretion system-mediated microbial competition is PhoPQ controlled and reactive oxygen species dependent
Source: PLoS Pathog. 2020 Mar 19;16(3):e1007969. doi: 10.1371/journal.ppat.1007969 (PMC7108748; doi:10.1371/journal.ppat.1007969)
Supplement: S14 Fig — Western blot analysis using anti-FLAG (A, B) and anti-VSV-G (C) antibodies demonstrating the presence of VgrG1, VgrG2, VgrG4, VgrG41-517, VgrG4570-899, VgrG4612-759, VgrG1603-750 in the cell lysates of E. coli. Membranes were reprobed with antibody anti RNA Polymerase α. Images are representative of three independent experiments. (PDF) [file ppat.1007969.s015.pdf]

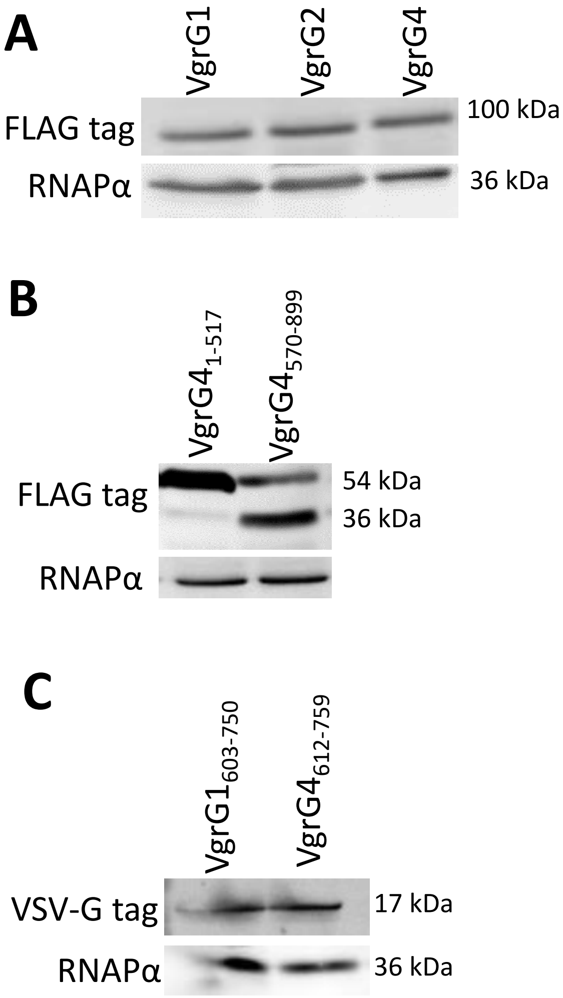

S14 Figure. Expression of VgrGs and truncated variants in *E. coli*.

Western blot analysis using anti-FLAG (A, B) and anti-VSV-G (C) antibodies demonstrating the presence of VgrG1, VgrG2, VgrG4, VgrG4$_{1\text{-}517}$, VgrG4$_{570\text{-}899}$, VgrG4$_{612\text{-}759}$, VgrG1$_{603\text{-}750}$ in the cell lysates of *E. coli*. Membranes were reprobed with antibody anti RNA Polymerase α. Images are representative of three independent experiments.
