# Supplementary material for: Klebsiella pneumoniae type VI secretion system-mediated microbial competition is PhoPQ controlled and reactive oxygen species dependent
Source: PLoS Pathog. 2020 Mar 19;16(3):e1007969. doi: 10.1371/journal.ppat.1007969 (PMC7108748; doi:10.1371/journal.ppat.1007969)
Supplement: S15 Fig — (A) Growth on SD (glucose) and SG (galactose) plates at 30°C and 37°C of serial dilutions of wild-type YPH499 yeast cells harbouring plasmids encoding the indicated vgrGs, and therefore expressing the corresponding proteins under the control of the GAL1 promoter on galactose-based medium. Image is representative of three independent experiments. (B) Immunoblot analysis of the expression of each of the VgrG4 variants in yeast using anti-GST antibody. (PDF) [file ppat.1007969.s016.pdf]

A

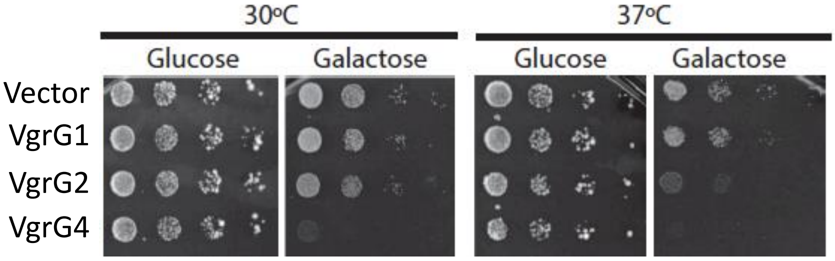

B

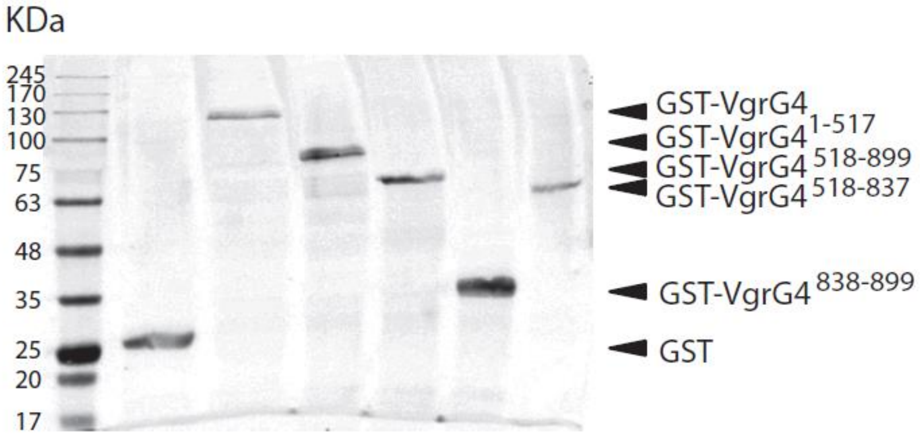

S15 Figure. VgrG4 is a trans-kingdom effector of *K. pneumoniae* T6SS.

(A) Growth on SD (glucose) and SG (galactose) plates at 30°C and 37^{0}C of serial dilutions of wild-type YPH499 yeast cells harbouring plasmids encoding the indicated *vgrGs*, and therefore expressing the corresponding proteins under the control of the GAL1 promoter on galactose-based medium. Image is representative of three independent experiments.

(B) Immunoblot analysis of the expression of each of the VgrG4 variants in yeast using anti-GST antibody.
