# Supplementary material for: Klebsiella pneumoniae type VI secretion system-mediated microbial competition is PhoPQ controlled and reactive oxygen species dependent
Source: PLoS Pathog. 2020 Mar 19;16(3):e1007969. doi: 10.1371/journal.ppat.1007969 (PMC7108748; doi:10.1371/journal.ppat.1007969)
Supplement: S16 Fig — (A) ROS levels in E. coli harbouring the indicated plasmids following induction of the Ara promoter of the pBAD plasmid with arabinose (0.1%) for 60 min by assessing the fluorescence of the dye CM-H2DCFDA. When indicated, ascorbate (15 mM) was added to supplement LB determined. Results are expressed in relative fluorescence units (RFU), and presented as means ± the standard deviations (n = 3). #, results are significantly different (P < 0.0001 [two-tailed t test]) from the results for bacteria harbouring pBAD30. (B) Single-cell analysis of ROS using the general ROS fluorescence sensor CM-H2DCFDA in E. coli harbouring pBADVgrG4570-699 pMMBSel1E following induction with arabinose and IPTG for 60 min. Hoechst was used to stain bacterial DNA. Images are representative of three independent experiments. When indicated, ascorbate (15 mM) was added to supplement the culture media. (C) Single-cell analysis of ROS using the general ROS fluorescence sensor CM-H2DCFDA in E. coli harbouring pBADVgrG4612-759 pMMBSel1E following induction with arabinose and IPTG for 60 min. Hoechst was used to stain bacterial DNA. Images are representative of three independent experiments. When indicated, ascorbate (15 mM) was added to supplement the culture media. (D) Single-cell analysis of ROS using the general ROS fluorescence sensor CM-H2DCFDA in E. coli upon co-cultivation with Kp52145, 52145-ΔclpV (ΔclpV), 52145-ΔvgrG4 (ΔvgrG4), 52145-ΔvgrG4 harbouring pBADVgrG4 (ΔvgrG4/ pBADVgrG4). Hoechst was used to stain bacterial DNA. Images are representative of three independent experiments. (E) Single-cell analysis of ROS using the general ROS fluorescence sensor CM-H2DCFDA in E. coli harbouring pMMBSel1E upon co-cultivation with Kp52145 following induction with IPTG for 60 min. Hoechst was used to stain bacterial DNA. Images are representative of three independent experiments. (PDF) [file ppat.1007969.s017.pdf]

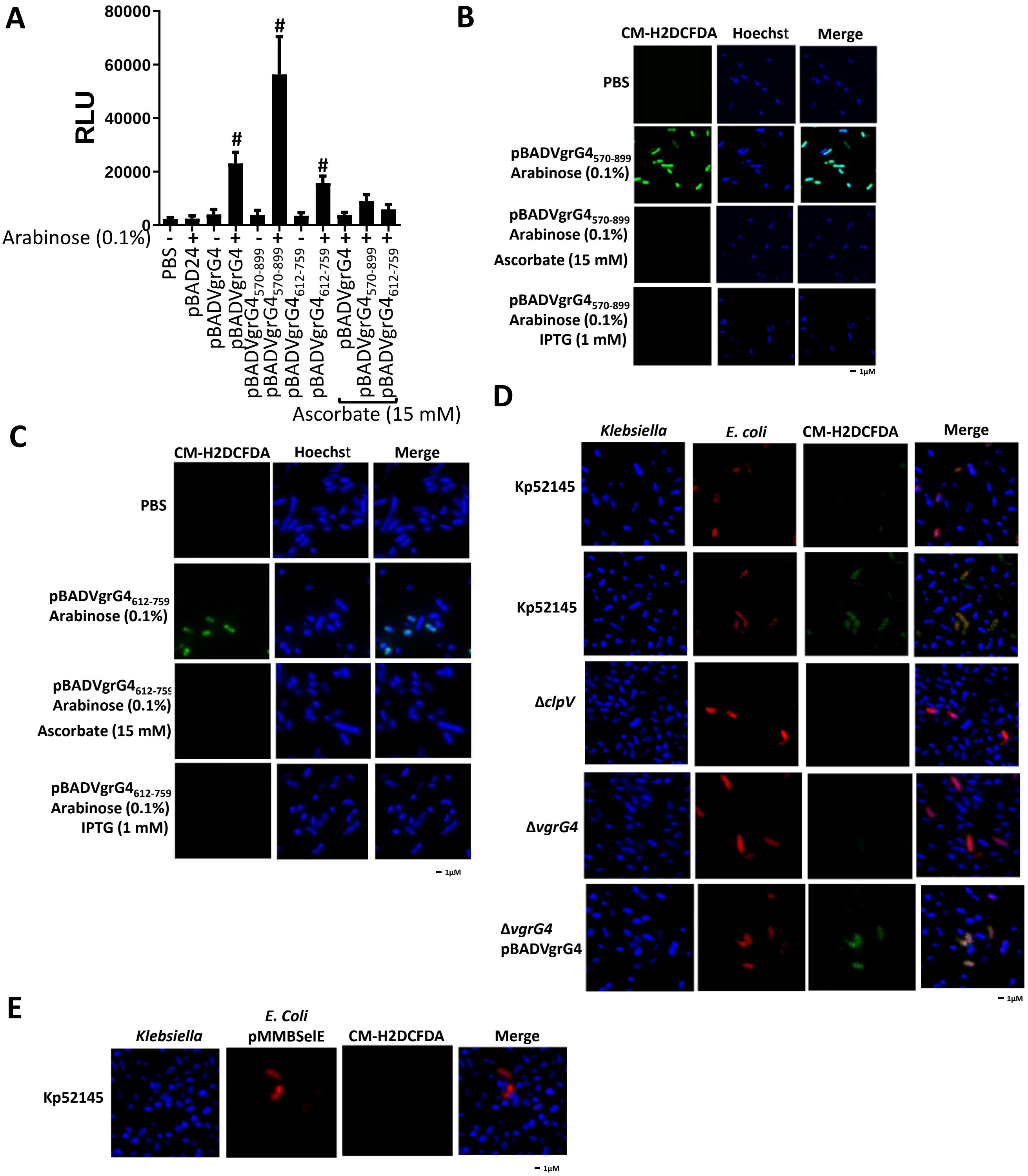

S16 Figure. VgrG4 toxic effect is ROS-dependent.

(A) ROS levels in *E. coli* harbouring the indicated plasmids following induction of the *Ara* promoter of the pBAD plasmid with arabinose (0.1%) for 60 min by assessing the fluorescence of the dye CM-H2DCFDA. When indicated, ascorbate (15 mM) was added to supplement LB determined. Results are expressed in relative fluorescence units (RFU), and presented as means ± the standard

deviations (n = 3). #, results are significantly different ($P < 0.0001$ [two-tailed t test]) from the results for bacteria harbouring pBAD30.

(B) Single-cell analysis of ROS using the general ROS fluorescence sensor CM-H2DCFDA in *E. coli* harbouring pBADVgrG4$_{570\text{-}699}$ pMMBSel1E following induction with arabinose and IPTG for 60 min. Hoechst was used to stain bacterial DNA. Images are representative of three independent experiments. When indicated, ascorbate (15 mM) was added to supplement the culture media.

(C) Single-cell analysis of ROS using the general ROS fluorescence sensor CM-H2DCFDA in *E. coli* harbouring pBADVgrG4$_{612\text{-}759}$ pMMBSel1E following induction with arabinose and IPTG for 60 min. Hoechst was used to stain bacterial DNA. Images are representative of three independent experiments. When indicated, ascorbate (15 mM) was added to supplement the culture media.

(D) Single-cell analysis of ROS using the general ROS fluorescence sensor CM-H2DCFDA in *E. coli* upon co-cultivation with Kp52145, 52145-Δ*clpV* (Δ*clpV*), 52145-Δ*vgrG4* (Δ*vgrG4*), 52145-Δ*vgrG4* harbouring pBADVgrG4 (Δ*vgrG4*/ pBADVgrG4). Hoechst was used to stain bacterial DNA. Images are representative of three independent experiments.

(E) Single-cell analysis of ROS using the general ROS fluorescence sensor CM-H2DCFDA in *E. coli* harbouring pMMBSel1E upon co-cultivation with Kp52145 following induction with IPTG for 60 min. Hoechst was used to stain bacterial DNA. Images are representative of three independent experiments.
