# Supplementary material for: Klebsiella pneumoniae type VI secretion system-mediated microbial competition is PhoPQ controlled and reactive oxygen species dependent
Source: PLoS Pathog. 2020 Mar 19;16(3):e1007969. doi: 10.1371/journal.ppat.1007969 (PMC7108748; doi:10.1371/journal.ppat.1007969)
Supplement: S17 Fig — A) Sel1E is not a general ROS scavenger. Single-cell analysis of ROS using the general ROS fluorescence sensor CM-H2DCFDA in E. coli harbouring pMMBSel1E upon incubation with ethanol (20%) following induction with IPTG for 60 min. Hoechst was used to stain bacterial DNA. Images are representative of three independent experiments. (B) soxS, oxyS, oxyR, and kat levels assessed by RT-qPCR in E. coli harbouring the indicated plasmids following induction of the Ara promoter of the pBAD plasmid with arabinose (0.1%), and the tac promoter of the pMMB207 plasmid with IPTG (1 mM) for 60 min. The data are presented as means ± the standard deviations (n = 3). (PDF) [file ppat.1007969.s018.pdf]

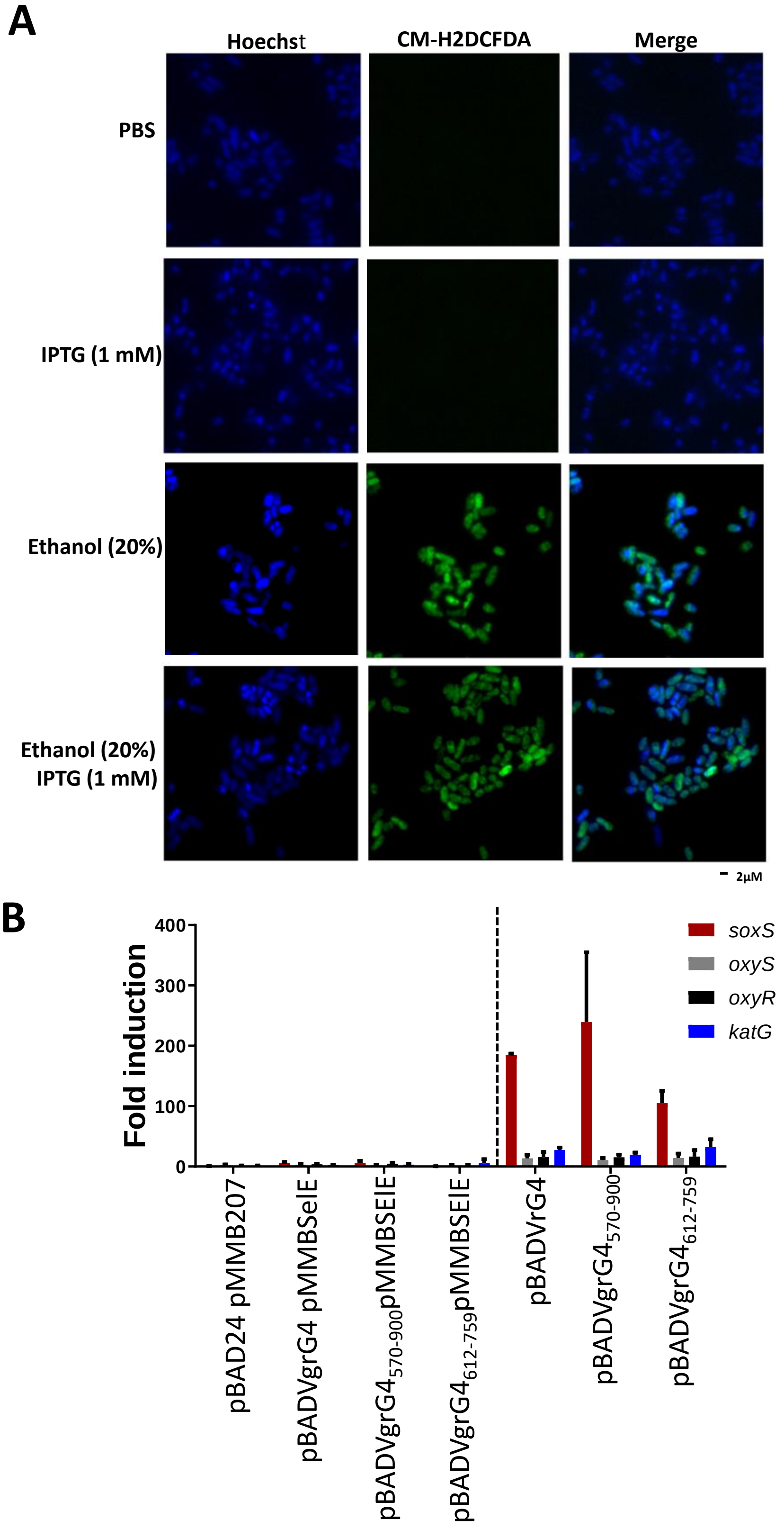

S17 Figure. Sel1E abolishes VgrG4-induced ROS responses.

(A) Sel1E is not a general ROS scavenger. Single-cell analysis of ROS using the general ROS fluorescence sensor CM-H2DCFDA in *E. coli* harbouring pMMBSel1E upon incubation with ethanol

(20%) following induction with IPTG for 60 min. Hoechst was used to stain bacterial DNA. Images are representative of three independent experiments.

(B) *soxS, oxyS, oxyR*, and *kat* levels assessed by RT-qPCR in *E. coli* harbouring the indicated plasmids following induction of the *Ara* promoter of the pBAD plasmid with arabinose (0.1%), and the *tac* promoter of the pMMB207 plasmid with IPTG (1 mM) for 60 min. The data are presented as means ± the standard deviations (n = 3).
